# Inclusion Membrane Growth and Composition Are Altered by Overexpression of Specific Inclusion Membrane Proteins in Chlamydia trachomatis L2

**DOI:** 10.1128/IAI.00094-21

**Published:** 2021-06-16

**Authors:** Macy G. Olson-Wood, Lisa M. Jorgenson, Scot P. Ouellette, Elizabeth A. Rucks

**Affiliations:** a Department of Pathology and Microbiology, University of Nebraska Medical Center, Omaha, Nebraska, USA; Yale University School of Medicine

**Keywords:** chlamydia, inclusion membrane, Inc, developmental cycle, type III secretion, *Chlamydia trachomatis*

## Abstract

Chlamydia trachomatis is the leading cause of bacterial sexually transmitted infections. This obligate intracellular bacterium develops within a membrane-bound vacuole called an inclusion, which sequesters the chlamydiae from the host cytoplasm. Host-pathogen interactions at this interface are mediated by chlamydial inclusion membrane proteins (Incs). However, the specific functions of most Incs are poorly characterized. Previous work from our laboratories indicated that expressing an IncF fusion protein at high levels in C. trachomatis L2 negatively impacted inclusion expansion and progeny production. We hypothesize that some Incs function in the structure and organization of the inclusion membrane and that overexpression of those Incs will alter the composition of endogenous Incs within the inclusion membrane. Consequently, inclusion biogenesis and chlamydial development are negatively impacted. To investigate this, C. trachomatis L2 was transformed with inducible expression plasmids encoding IncF-, CT813-, or CT226-FLAG. Overexpression of IncF-FLAG or CT813-FLAG, but not CT226-FLAG, altered chlamydial development, as demonstrated by smaller inclusions, fewer progeny, and increased plasmid loss. The overexpression of CT813-FLAG reduced the detectable levels of endogenous IncE and IncG in the inclusion membrane. Notably, recruitment of sorting nexin-6, a eukaryotic protein binding partner of IncE, was also reduced after CT813 overexpression. Gene expression studies and ultrastructural analysis of chlamydial organisms demonstrated that chlamydial development was altered when CT813-FLAG was overexpressed. Overall, these data indicate that disrupting the expression of specific Incs changed the composition of Incs within the inclusion membrane and the recruitment of associated host cell proteins, which negatively impacted C. trachomatis development.

## INTRODUCTION

The obligate intracellular bacterium Chlamydia trachomatis is the most common bacterial sexually transmitted infection and the leading cause of preventable infectious blindness worldwide (1). After the infectious elementary body (EB) is internalized into a susceptible host cell, the EB differentiates into a reticulate body (RB) and develops within a membrane-bound vacuole, termed the inclusion. The nascent inclusion, derived from a eukaryotic vacuole, is extensively modified by chlamydial proteins (2–5). The inclusion membrane acts as the host-pathogen interface and mediates necessary interactions with the host cell while physically separating the chlamydial organisms from the cytosol. Chlamydial protein synthesis is required to prevent inclusion-lysosome fusion (6, 7) and to direct the inclusion to the microtubule organizing center (MTOC) (2, 3, 8). Moreover, the inclusion expands during the developmental cycle, independent of bacterial replication but dependent on active chlamydial protein synthesis (9). Blocking chlamydial protein synthesis with chloramphenicol also inhibits the expansion of the inclusion (9), the secretion of type III secreted effectors, and the incorporation of host lipids into the inclusion membrane (3, 7, 10–14), indicating that chlamydial proteins and processes play a fundamental role in the biogenesis of the inclusion membrane. Thus, the composition and structural integrity of the inclusion membrane are essential for chlamydial development (15).

The inclusion membrane is modified primarily by a class of type III secreted effectors called inclusion membrane (Inc) proteins (16–19). Incs are characterized as containing two or more hydrophobic transmembrane domains (17, 20, 21) with both the N and C termini predicted to be exposed to the host cytosol (22). For C. trachomatis, there are 60 to 80 predicted *inc* genes, which is approximately 7% to 8% of the highly reduced chlamydial genome (17, 23). The retention of a large percentage of predicted *inc* genes supports a critical role for Incs in intracellular survival (24). In addition, Incs are temporally expressed throughout the developmental cycle (25, 26), suggesting that their expression is associated with their function in the inclusion membrane. For example, the transcription of some *inc* genes, like *incF*, is detected early in the developmental cycle (27). Other *inc* genes, such as *incA*, are not transcribed until later (i.e., mid-developmental cycle) (25, 26). However, the specific function of most Incs remains unknown (24, 28). Some Incs facilitate interactions between different host cell compartments to acquire nutrients that are important both for the growing inclusion and for chlamydial development (2, 29–32). Some Inc proteins may function in inclusion stability by (i) recruiting a eukaryotic protein that contributes to the structural integrity of the inclusion (e.g., actin [33]), (ii) organizing the localization of other Incs, or (iii) contributing to the balanced Inc protein composition of the inclusion membrane. The loss of any of these functions may negatively impact inclusion development. Support for this concept comes from Inc-eukaryotic protein-protein interactions involved in the modification of the host cytoskeleton, vesicle trafficking, and lipid acquisition (34–39), processes that have been proposed to facilitate the expansion of the inclusion (15). Highlighting the importance of balanced Inc composition in the inclusion membrane is the observation that both the loss of certain Incs (e.g., CT229, CT383, and IncC) (15) and the overexpression of certain Incs (e.g., IncF-APEX2) (40) can result in a phenotype of reduced inclusion size.

The inclusion must be remodeled by chlamydial proteins for the intracellular survival of chlamydiae (9). Thus, the secretion and abundance of Inc proteins are likely tightly regulated. For example, too much of a specific Inc in the inclusion membrane may cause that Inc to be mislocalized, which may negatively impact the appropriate insertion and/or localization of other endogenous Incs. Restricted inclusion expansion upon the overexpression of certain Incs may be a phenotypic indicator of disrupted Inc composition in the inclusion membrane. We hypothesize that some Incs function in the structure and organization of the inclusion membrane. The overexpression of such Incs is predicted to alter the composition of endogenous Incs in the inclusion membrane, negatively impacting inclusion biogenesis and chlamydial development. In support of this, the reduced inclusion expansion upon high levels of IncF-APEX2 expression may suggest a function for IncF in the structural integrity or in the organization of Incs in the inclusion membrane (40). Consistent with this hypothesis, IncF has been implicated as a hub of Inc-Inc protein-protein interactions based on bacterial two-hybrid analyses (BACTH) (28). Data from a recent study in our lab showed that the eukaryotic host protein VAMP3 interacted with four different Incs between 15 and 23 h postinfection (hpi), with IncF being the first of these Incs (41), and the second Inc, IncG, being a possible IncF interactor by BACTH (28). These data suggest that IncF may also be a hub for eukaryotic proteins that can interact with multiple Incs (41).

To better understand the phenotype that we originally observed with the overexpression of IncF-APEX2, we used the overexpression of different Inc-FLAG proteins from C. trachomatis L2 as a tool to evaluate the function of Inc proteins and the impact of Inc abundance on the composition of the inclusion membrane and on chlamydial development. For this study, we chose to evaluate and compare two mid-cycle Incs, CT813 (also referred to as InaC [42]) and CT226, with IncF. By BACTH, CT813 demonstrates homotypic interactions (28), whereas CT226 binds multiple Incs, similar to IncF (43). High expression levels of CT813 and IncF, but not CT226, negatively impacted growth of the bacteria and resulted in the loss of selected endogenous Incs (e.g., IncE) in the inclusion membrane. Increased expression of CT813 also reduced the recruitment of sorting nexin-6 (SNX6), a eukaryotic protein binding partner of IncE, to the inclusion during infection. Finally, overexpression of CT813 altered developmental cycle progression, as determined by transcript and ultrastructural analyses of chlamydial developmental forms. This study demonstrates that altering Inc expression levels can have negative consequences for inclusion membrane expansion and chlamydial development.

## RESULTS

### Decreased inclusion expansion and production of infectious progeny after overexpression of CT813-FLAG and IncF-FLAG from C. trachomatis L2.

To determine if the overexpression of certain Incs negatively impacts chlamydial development, C. trachomatis L2 was transformed with anhydrotetracycline (aTc)-inducible plasmids encoding CT813-FLAG, IncF-FLAG, CT226-FLAG, CT483-FLAG, or mCherry (empty vector control) (44). Inclusion size was used as a metric for normal chlamydial development. CT483 was a predicted Inc protein based on the presence of transmembrane domains, but CT483-FLAG was observed to localize to the membrane of chlamydiae and not to the inclusion membrane (23). Therefore, the inducible expression of CT483-FLAG was used as a control for the metabolic burden of overproducing a membrane protein in C. trachomatis L2 transformed strains. The C. trachomatis L2 transformed strains were induced for expression using increasing concentrations of aTc to drive increasing construct expression (i.e., 1 nM, 5 nM, or 20 nM aTc), and inclusion size was examined.

**FIG 1:**
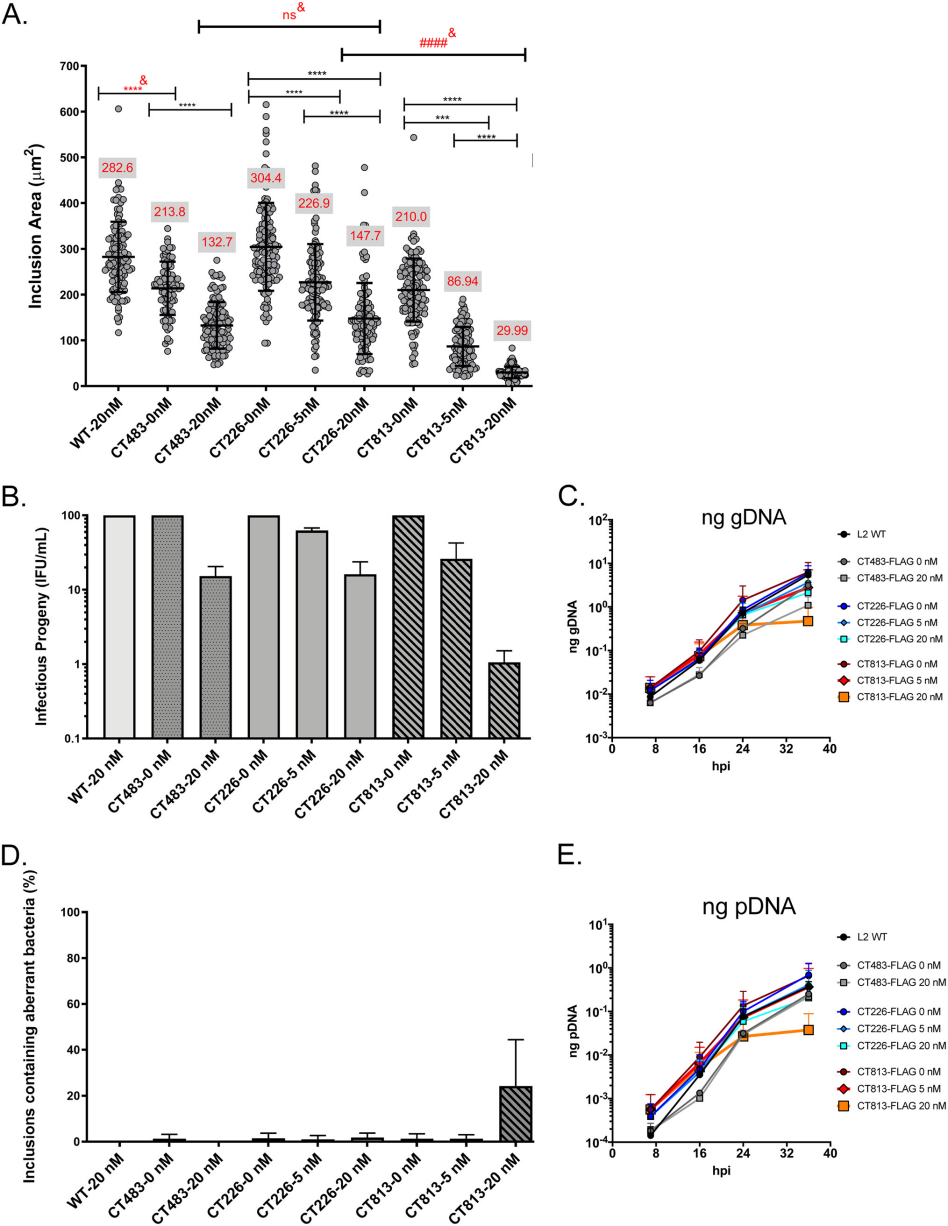
Overexpression of CT813-FLAG from C. trachomatis L2 negatively impacts inclusion growth and progeny production. (A) HeLa cells seeded on coverslips were infected with C. trachomatis L2 CT813-FLAG, CT226-FLAG, or CT483-FLAG transformed strains or wild-type L2 and induced at 7 hpi with 5 or 20 nM aTc or not induced. Coverslips were methanol fixed at 36 hpi and stained for immunofluorescence to determine the inclusion area. The inclusion areas (μm^2^) with standard deviations were plotted and analyzed for statistical significance by an ordinary one-way ANOVA with Tukey’s multiple-comparison test using GraphPad Prism 8.4.0. These data are combined from three biological replicates. ####^&^ indicates a significant difference between C. trachomatis L2 transformed strains induced with 20 nM aTc. ****^&^, *P* < 0.0001; ***, *P* = 0.0004; all *P* values reflect comparison to the control (wild-type and uninduced strains); ns, not significant. The red values in the gray box in panel A are the average inclusion area for each *C. trachomatis* L2 transformed strain. (B) Duplicate wells of HeLa cells were infected as described for panel A. At 36 hpi, infected monolayers were lysed, serially diluted, and infected onto a fresh monolayer of HeLa cells (i.e., secondary infection) in medium containing penicillin to enumerate infectious progeny (inclusion-forming units [IFU]/ml). Infectious progeny (IFU/ml) (normalized to uninduced strains and expressed as a percentage of uninduced from three biological replicates and standard deviation) were plotted and statistically analyzed (ordinary one-way ANOVA with Tukey’s multiple-comparison test using GraphPad Prism 8.4.0). Only inclusions containing normal (not aberrant) bacteria were enumerated. (C and E) HeLa cells were infected with C. trachomatis L2 CT813-FLAG, CT226-FLAG, or CT483-FLAG transformed strains, or wild-type C. trachomatis L2, and either not induced or induced at 7 hpi (5 nM or 20 nM aTc). DNA collected from separate wells of a 6-well plate at 7, 16, 24, and 36 hpi was processed as described in Materials and Methods. The genomic DNA (gDNA; ng) (C) and plasmid DNA (pDNA; ng) (E) data sets from three biological replicates were plotted using GraphPad Prism 8.4.0. The data are representative of three independent experiments. (D) Plasmid loss was indicated by inclusions containing aberrant bacteria in medium containing penicillin (i.e., sensitivity due to the loss of the plasmid-borne *bla* resistance gene). To enumerate the percentage of inclusions containing aberrant bacteria, the number of inclusions with aberrant bacteria was divided by the total number of inclusions counted (B) from three biological replicates and the standard deviation was plotted and statistically analyzed as described for panels A and B using GraphPad Prism 8.4.0. Differences were not statistically significant.

HeLa cells seeded on coverslips were infected with the C. trachomatis L2 CT813-FLAG, CT226-FLAG, IncF-FLAG, or CT483-FLAG transformed strains, or wild-type C. trachomatis L2, and induced for expression with aTc as indicated, or not induced, at 7 h postinfection (hpi). Coverslips were fixed at 36 hpi and processed for indirect immunofluorescence using an anti-FLAG antibody to confirm construct expression, an anti-IncA antibody to label the inclusion membrane, and an anti-MOMP (major outer membrane protein) antibody to indicate Chlamydia, and inclusion area was measured (Fig. 1A; see Fig. S1A in the supplemental material). At 36 hpi, the mean inclusion area for wild-type C. trachomatis L2 was 282.6 ± 76.9 μm^2^ (Fig. 1A). The mean inclusion areas were 213.8 ± 58.3 μm^2^ and 132.7 ± 50.9 μm^2^ for uninduced (0 nM) and 20 nM aTc-treated C. trachomatis L2 CT483-FLAG, respectively (Fig. 1A). The inclusion area of C. trachomatis L2 CT226-FLAG not induced for expression was 304 ± 96.1 μm^2^, while the induction of CT226-FLAG using 5 nM and 20 nM aTc resulted in mean inclusion areas of 226.9 ± 83.1 μm^2^ and 147.7 ± 77.7 μm^2^, respectively. The inclusion areas for C. trachomatis L2 CT483-FLAG and CT226-FLAG induced for expression using 20 nM aTc were not significantly different, which may suggest that the changes in inclusion area reflect the metabolic burden of construct expression.

Consistent with our prior study using C. trachomatis L2 IncF-APEX2 (40), the overexpression of IncF-FLAG from C. trachomatis L2 resulted in significantly smaller inclusions (5 or 20 nM aTc) (Fig. S1A). The inclusion areas of C. trachomatis L2 IncF-FLAG were 247.0 ± 58.8 μm^2^ for uninduced cultures, 171.4 ± 59.0 μm^2^ for 1 nM aTc, 57.66 ± 18.1 μm^2^ for 5 nM aTc, and 27.5 ± 15.5 μm^2^ for 20 nM (Fig. S1A). These data indicate that the size of APEX2 (27 kDa [45] compared to an ∼1-kDa FLAG epitope tag) did not contribute to the defects associated with overexpression of IncF. Of note, induction by greater concentrations of aTc was tolerated in the strain expressing IncF-FLAG versus IncF-APEX2 (data not shown) (40). Similarly, the overexpression of an epitope-tagged CT813-FLAG (Fig. 1A) and untagged CT813 (Fig. S1B) from C. trachomatis L2 transformed strains each resulted in visibly smaller inclusions, which further supports that the observed phenotype is not due to the presence of the FLAG tag. The mean inclusion area for C. trachomatis L2 CT813-FLAG not induced for expression was 210.0 ± 68.9 μm^2^, compared to 86.94 ± 42.7 μm^2^ using 5 nM aTc and 29.99 ± 12.5 μm^2^ using 20 nM aTc (Fig. 1A). The mean inclusion areas for each C. trachomatis L2 CT813-FLAG and IncF-FLAG strain induced with 20 nM aTc were both significantly smaller than the mean inclusion area for C. trachomatis L2 CT226-FLAG strain induced using 20 nM aTc (Fig. 1A; Fig. S1A and B). Reducing the induction conditions (i.e., aTc) moderately restored inclusion expansion for C. trachomatis L2 CT813-FLAG and IncF-FLAG transformants (Fig. 1A; Fig. S1A). Finally, the constitutive expression of mCherry from C. trachomatis L2 transformed with pBOMB-mCherry (44) also did not significantly impact inclusion size (Fig. S1B). Thus, smaller inclusions may be an indicator of altered or dysregulated inclusion membrane composition due to the overabundance of certain Incs, such as CT813 and IncF.

The impact of the overexpression of the Inc-FLAG constructs on the production of infectious progeny was also examined. HeLa cells were infected with C. trachomatis L2 transformed strains or wild-type L2 and induced or not using aTc as described above. At 36 hpi, C. trachomatis L2-infected HeLa cell monolayers were lysed, serially diluted, and plated onto a fresh HeLa cell monolayer to enumerate infectious progeny. The secondary infection assays were performed in medium containing penicillin to distinguish inclusions containing Chlamydia that retained the plasmid (encoding beta-lactamase) from those that lost the plasmid (i.e., morphologically aberrant Chlamydia susceptible to penicillin). Consistent with the decreased inclusion area, the overexpression of CT813-FLAG or IncF-FLAG from C. trachomatis L2 transformed strains also resulted in fewer infectious progeny than the overexpression of CT226-FLAG or CT483-FLAG (Fig. 1B). Compared to uninduced samples, C. trachomatis L2 CT483-FLAG induced with 20 nM aTc resulted in a 5.2-fold decrease in infectious progeny. CT226-FLAG expression (20 nM aTc) resulted in a 4.6-fold decrease compared to uninduced samples (Fig. 1B). For C. trachomatis L2 CT813-FLAG cultures induced with 20 nM aTc compared to uninduced samples, there was a 35.3-fold decrease in infectious progeny (Fig. 1B). Similarly, for C. trachomatis L2 IncF-FLAG cultures induced with 20 nM aTc compared to uninduced samples, an 81.6-fold decrease in infectious progeny was observed (Fig. S1C). By quantitative PCR (qPCR), C. trachomatis L2 CT813-FLAG induced using 20 nM aTc had reduced detectable genomic DNA (gDNA) (Fig. 1C, orange line), consistent with the decrease in infectious progeny that were enumerated.

The secondary infection assay also revealed an increase in inclusions derived from bacteria that had lost the expression plasmid (i.e., harboring aberrant bacteria) upon the overexpression of CT813-FLAG or IncF-FLAG, but not CT226-FLAG or CT483-FLAG, from the C. trachomatis L2 transformed strains (Fig. 1D). Only about 3% of the inclusions harbored aberrant bacteria for C. trachomatis L2 CT226-FLAG and CT483-FLAG strains that had been induced (with 20 nM aTc) for expression in the primary infection (Fig. 1D). However, the overexpression of CT813-FLAG or IncF-FLAG from C. trachomatis L2 transformed strains that were induced with 20 nM aTc (in the primary infection) resulted in approximately 25% of the inclusions from C. trachomatis L2 CT813-FLAG (Fig. 1D) and 50% of the inclusions from C. trachomatis L2 IncF-FLAG harboring aberrant bacteria (Fig. S1D). Plasmid loss was abrogated when lower induction conditions were used during the primary infection (i.e., 1 or 5 nM aTc) with these strains (Fig. 1D; Fig. S1D). Plasmid loss was also observed by qPCR, where C. trachomatis L2 CT813-FLAG cultures induced with 20 nM aTc had reduced detectable plasmid DNA (Fig. 1E, orange line). Overall, these data indicate that the overexpression of CT813-FLAG or IncF-FLAG negatively impacted chlamydial development, as demonstrated by decreased inclusion expansion, fewer infectious progeny, reduced genomic DNA levels, and plasmid loss with subsequent sensitivity to penicillin, the selecting agent.

### Loss of detectable IncA, CT223, and IncE by Western blotting after overexpression of CT813-FLAG, but not CT226-FLAG, from C. trachomatis L2 transformed strains.

**FIG 2:**
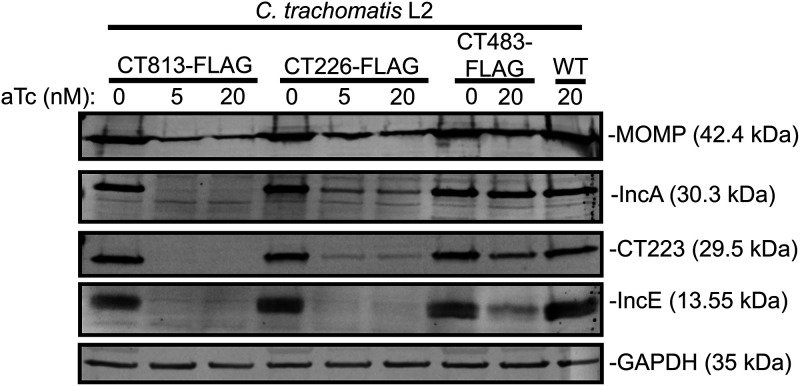
The overexpression of CT813-FLAG from C. trachomatis L2 results in decreased detectable IncA, CT223, and IncE. HeLa cells were infected with C. trachomatis L2 CT813-FLAG, CT226-FLAG, or CT483-FLAG transformed strains, or wild-type C. trachomatis L2, and either not induced or induced at 7 hpi (5 nM or 20 nM aTc). At 48 hpi, infected monolayers were lysed in 8 M urea supplemented with 1% SDS, 10 mM Tris-HCl (pH 7.4), 2.5% β-mercaptoethanol, and nuclease. Lysates were normalized for equal protein content, separated by SDS-PAGE, and transferred to PVDF to blot for chlamydial proteins, MOMP, IncA, CT223, and IncE. GAPDH was used as a loading control. These data are representative of three independent experiments.

Next, we examined if the negative impact on chlamydial development was a consequence of altered Inc composition by examining endogenous Inc expression. However, these studies are limited by the Incs for which we have antibodies. We therefore determined the levels of endogenous IncE, IncA, and CT223 in infected cell lysates by Western blotting. For these studies, we primarily used the CT813-FLAG overexpression strain, as the chlamydial strain transformed with pBOMB-*incF_FLAG* demonstrates “leaky expression” when no aTc is added to cultures (43). HeLa cells seeded in a 6-well plate containing a glass coverslip in each well were infected with C. trachomatis L2 CT813-FLAG, CT226-FLAG, or CT483-FLAG transformed strains or with wild-type C. trachomatis L2 and were induced, or not, at 7 hpi using 5 or 20 nM aTc. Coverslips were included to confirm construct expression via indirect immunofluorescence, since detection of the Inc-FLAG constructs is not always feasible by Western blotting the total lysate. However, we have previously shown that Inc-FLAG expression can be observed by affinity purification with anti-FLAG beads (43). Cell lysates were collected at 36 hpi (Fig. S2) and 48 hpi (Fig. 2), and equal amounts of proteins were analyzed across samples (see Materials and Methods). C. trachomatis L2 MOMP was detected for each C. trachomatis L2 strain at each aTc concentration, although MOMP was decreased to various degrees when each C. trachomatis L2 transformed strain was overexpressed (Fig. 2; Fig. S2). In addition, CT813-FLAG (30.5 kDa) and CT226-FLAG (19.2 kDa) induced for expression (5 and 20 nM aTc) were detected at the expected molecular weights (Fig. S2). CT483-FLAG (13.8 kDa) was not observed (Fig. S2), which may be attributed to the lower abundance of this protein in total lysate. When using the same FLAG antibody that was used for Western blotting, CT483-FLAG can be detected by indirect immunofluorescence, which indicates that CT483-FLAG is being expressed as expected. The overexpression of CT813-FLAG using 5 or 20 nM aTc resulted in the loss of detectable IncA (30.3 kDa), CT223 (29.5 kDa), and IncE (13.55 kDa) by Western blotting (Fig. 2; Fig. S2). The overexpression of CT226-FLAG or CT483-FLAG from C. trachomatis L2 transformed strains, using 5 or 20 nM aTc, reduced detectable IncA, CT223, and IncE (Fig. 2) but not to the same extent as observed after overexpression of CT813-FLAG (Fig. 2; Fig. S2).

### Altered localization of endogenous Incs in the inclusion membrane after overexpression of CT813-FLAG and IncF-FLAG from C. trachomatis L2 transformed strains.

As chlamydial proteins are less abundant than eukaryotic proteins in infected cells, and hydrophobic Inc proteins are difficult to effectively solubilize, Inc protein levels may be below the limit of detection of a particular assay (43). Further, Western blot analysis alone does not demonstrate if Incs are within the organisms or inserted in the inclusion membrane. To determine if the overexpression of certain Inc-FLAG constructs impacted the ability to detect endogenous Incs in the inclusion membrane, we assessed the localization and intensity of endogenous Incs by an indirect immunofluorescence assay. HeLa cells seeded on coverslips were infected with C. trachomatis CT813-FLAG, IncF-FLAG, CT226-FLAG, or CT483-FLAG transformed strains, or with wild-type L2, and were induced or not using 5 or 20 nM aTc at 7 hpi. At 36 hpi, the coverslips were methanol fixed and processed for immunofluorescence using anti-IncE, -IncA, -IncG, and -CT223 antibodies to observe the expression and localization of these endogenous Incs. Coverslips were imaged using the same exposure time to compare the intensities of endogenous Incs between the different C. trachomatis L2 strains and induction conditions used.

**FIG 3:**
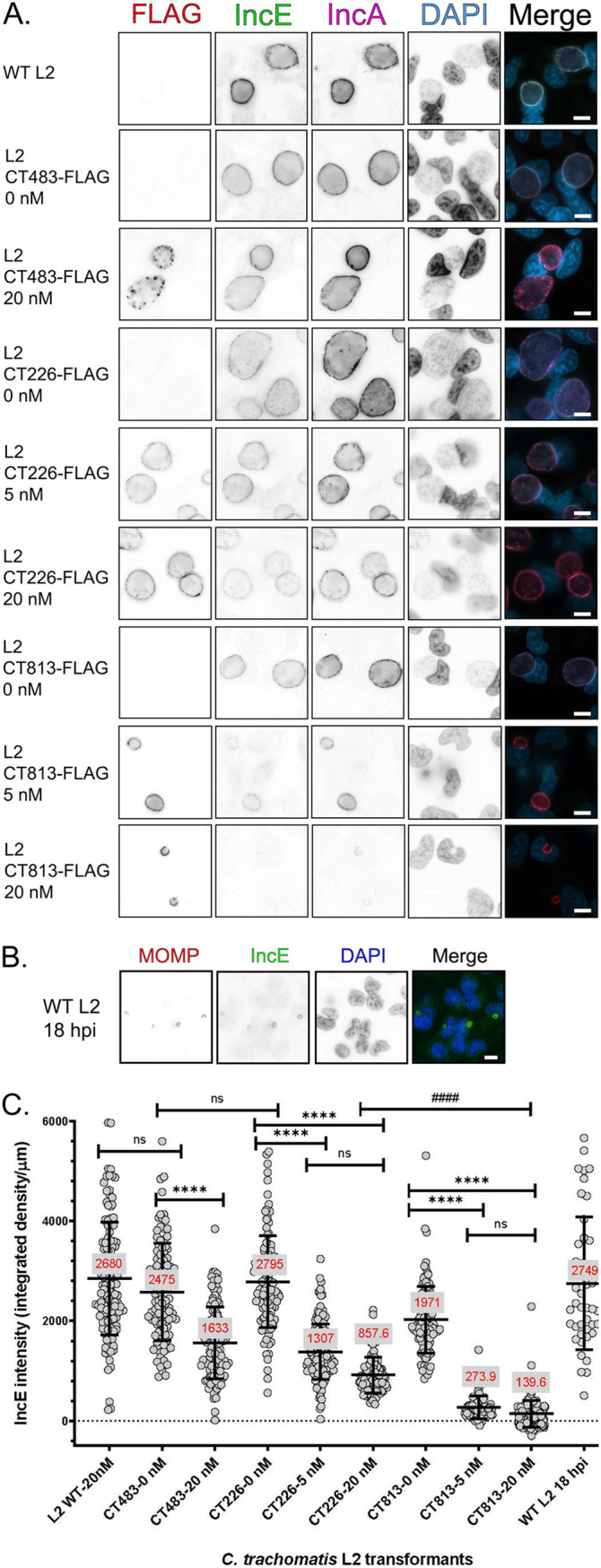
The overexpression of CT813-FLAG from C. trachomatis L2 results in loss of detectable endogenous IncE in the inclusion membrane. (A) HeLa cells infected with C. trachomatis L2 transformed strains or wild-type L2 were induced with 5 or 20 nM aTc or not induced at 7 hpi. Coverslips were methanol fixed at 36 hpi and stained for immunofluorescence to observe expression of the constructs (FLAG; red), IncE (green), IncA (pink), or DNA (DAPI; blue) and imaged at ×63 magnification using the same exposure for each sample. Scale bar, 10 μm. Individual panels were converted to black and white and inverted to visualize the loss of IncE staining. These data are from three biological replicates. (B) C. trachomatis L2-infected HeLa cells were fixed at 18 hpi and stained for immunofluorescence to observe IncE (green), MOMP (red), and DNA (blue). Coverslips were imaged using the same exposure for each sample at ×63 magnification. Scale bar, 10 μm. (C) The intensity of IncE was measured using ImageJ from a minimum of 80 inclusions per experiment (see Materials and Methods). For each image, the background integrated density was subtracted from individual images, and the intensity was normalized to the inclusion perimeter (integrated density/μm). The mean integrated density/μm is reported in red for each sample measured. The intensity (integrated density/μm) and standard deviation were plotted using GraphPad Prism 8.4.0. Samples were analyzed for statistical significance using a one-way ANOVA and Tukey’s multiple-comparison test. ****, *P* < 0.0001 between C. trachomatis L2 transformed strains; ####, *P* < 0.0001 between C. trachomatis L2 transformed strains.

By indirect immunofluorescence assay, wild-type L2 inclusions demonstrated strong IncE and IncA staining at the inclusion membrane (Fig. 3A). Inducing the expression of CT483-FLAG with 20 nM aTc from C. trachomatis L2 CT483-FLAG resulted in detection of CT483-FLAG within the chlamydial organisms (Fig. 3A), consistent with previous findings (23). In addition, the overexpression of CT483-FLAG did not dramatically impact IncE or IncA levels in the inclusion membrane (Fig. 3A). The overexpression of CT226-FLAG with 20 nM aTc slightly reduced the presence of IncE in the inclusion membrane (Fig. 3A), which was consistent with the Western blot data (Fig. 2). However, the overexpression of CT813-FLAG with 20 nM aTc from C. trachomatis L2 CT813-FLAG resulted in the loss of detectable IncE and the near loss of IncA labeling (Fig. 3A). IncE was not detected at IncF-FLAG-positive inclusions that had been induced with either 5 or 20 nM aTc (Fig. S3). Notably, IncE was detected in the inclusion membranes of inclusions formed by wild-type C. trachomatis L2 at an earlier time point (18 hpi), which are similar in size to inclusions at 36 hpi from C. trachomatis L2 CT813-FLAG or IncF-FLAG induced with 20 nM aTc (Fig. 3B). These data suggest that the lack of IncE in the inclusion membranes of organisms overexpressing CT813-FLAG or IncF-FLAG is not due to a limitation of inclusion size or delayed development.

The intensity of IncE was quantified using ImageJ to measure the integrated density of IncE in individual inclusions. The IncE intensity was normalized to the inclusion perimeter to account for differences in inclusion size upon induction of construct expression (integrated density/μm). For example, inclusions formed by wild-type C. trachomatis L2 at 36 hpi and 18 hpi had similar IncE intensities (Fig. 3C). The IncE intensity of inclusions from C. trachomatis L2 CT483-FLAG induced with 20 nM aTc was decreased 1.5-fold compared to that of uninduced conditions (Fig. 3C). Similarly, using C. trachomatis L2 CT226-FLAG induced with 5 or 20 nM aTc, the intensity of IncE was decreased 2.1-fold and 3.3-fold, respectively, compared to the uninduced condition (Fig. 3C). In contrast, the IncE intensity of C. trachomatis L2 CT813-FLAG inclusions was significantly reduced, 7.2-fold and 14.1-fold, under inducing conditions with either 5 or 20 nM aTc, respectively, compared to uninduced conditions (Fig. 3C). The intensity of IncE when CT813-FLAG was overexpressed was also significantly different than the IncE intensity measured when CT226-FLAG was overexpressed (Fig. 3C).

The overexpression of CT813-FLAG, but not CT226-FLAG, also resulted in a similar loss in detectable IncG in the inclusion membrane (Fig. S4A), albeit to a lesser extent than observed for IncE (Fig. 3C). The anti-IncG antibody exhibits increased background staining in our hands compared to the anti-IncE antibody, which affects the accurate quantification of IncG intensity. The intensities of IncG from wild-type inclusions at both 36 hpi (Fig. S4A) and 18 hpi (Fig. S4B) were similar (Fig. S4C). A 1.73-fold decrease in IncG intensity was observed upon the overexpression of CT483-FLAG from C. trachomatis L2 CT483-FLAG with 20 nM aTc compared to the uninduced control (Fig. S4C). For C. trachomatis L2 CT226-FLAG induced with either 5 or 20 nM aTc, the intensity of IncG decreased 1.53-fold and 1.93-fold, respectively, compared to uninduced conditions (Fig. S4C). When CT813-FLAG was induced with either 5 or 20 nM aTc compared to uninduced conditions, the intensity of IncG decreased 2.99-fold and 4.41-fold, respectively (Fig. S4C). The intensity of endogenous IncA (Fig. S4D) was also decreased upon the overexpression of CT813-FLAG but not CT226-FLAG. There was a 1.12-fold increase in IncA intensity for C. trachomatis L2 CT483-FLAG with 20 nM aTc compared to uninduced conditions (Fig. S4D). For C. trachomatis L2 CT226-FLAG induced with either 5 or 20 nM aTc, the intensity of IncA decreased 1.09-fold and 1.23-fold, respectively, compared to uninduced conditions (Fig. S4D). When CT813-FLAG was induced with 5 or 20 nM aTc compared to uninduced conditions, the intensity of IncA decreased 4.89-fold and 8.43-fold, respectively (Fig. S4D). The localization of endogenous CT223 was also evaluated. By indirect immunofluorescence, the overexpression of the CT483-FLAG or CT226-FLAG from the respective C. trachomatis L2 transformed strains did not impact endogenous CT223 localization (Fig. S5). When L2 CT813-FLAG was overexpressed, CT223 was reduced at the inclusion membrane (Fig. S5). Consistent with previous publications (23, 46, 47), endogenous CT223 remained in microdomains around the inclusion upon overexpression of the C. trachomatis transformed strains (Fig. S5).

Finally, endogenous *ct813* is expressed mid-developmental cycle (26), so the impact of CT813-FLAG induction during mid-developmental cycle (i.e., 14.5 hpi) on IncE, IncG, and IncA intensity at the inclusion membrane was also examined. As above, the induction of CT813-FLAG from the C. trachomatis L2 CT813-FLAG transformant at 14.5 hpi with either 5 or 20 nM aTc resulted in visibly smaller inclusions than those of the uninduced control (Fig. S6). The overexpression of CT813-FLAG at 14.5 hpi also resulted in the loss of detectable IncE, IncG, and IncA at the inclusion membrane at 36 hpi (Fig. S6). Of note, inclusions harboring bacteria that did not retain the plasmid (i.e., morphologically aberrant Chlamydia susceptible to penicillin), which do not express the Inc-FLAG construct when induced, displayed strong IncG and IncA staining (Fig. S6B, asterisk). Overall, these data demonstrate that overexpression of CT813-FLAG at either 7 hpi or 14.5 hpi negatively impacts the presence of endogenous IncE, IncG, and IncA in the inclusion membrane.

The decrease in IncE, IncG, and IncA intensity in the inclusion membrane when CT813-FLAG is overexpressed, but not when CT226-FLAG is overexpressed, from C. trachomatis L2 transformed strains supports our hypothesis that the overexpression of certain Incs alters the composition of Incs in the inclusion membrane. These data may also support different functions for Incs, since the overexpression of CT226-FLAG does not considerably impact the localization or expression of endogenous Incs in the inclusion membrane, as determined by indirect immunofluorescence or by Western blotting, respectively.

### Decreased localization of sorting nexin-6, an IncE binding partner, at the inclusion membrane upon overexpression of CT813-FLAG from C. trachomatis L2.

**FIG 4:**
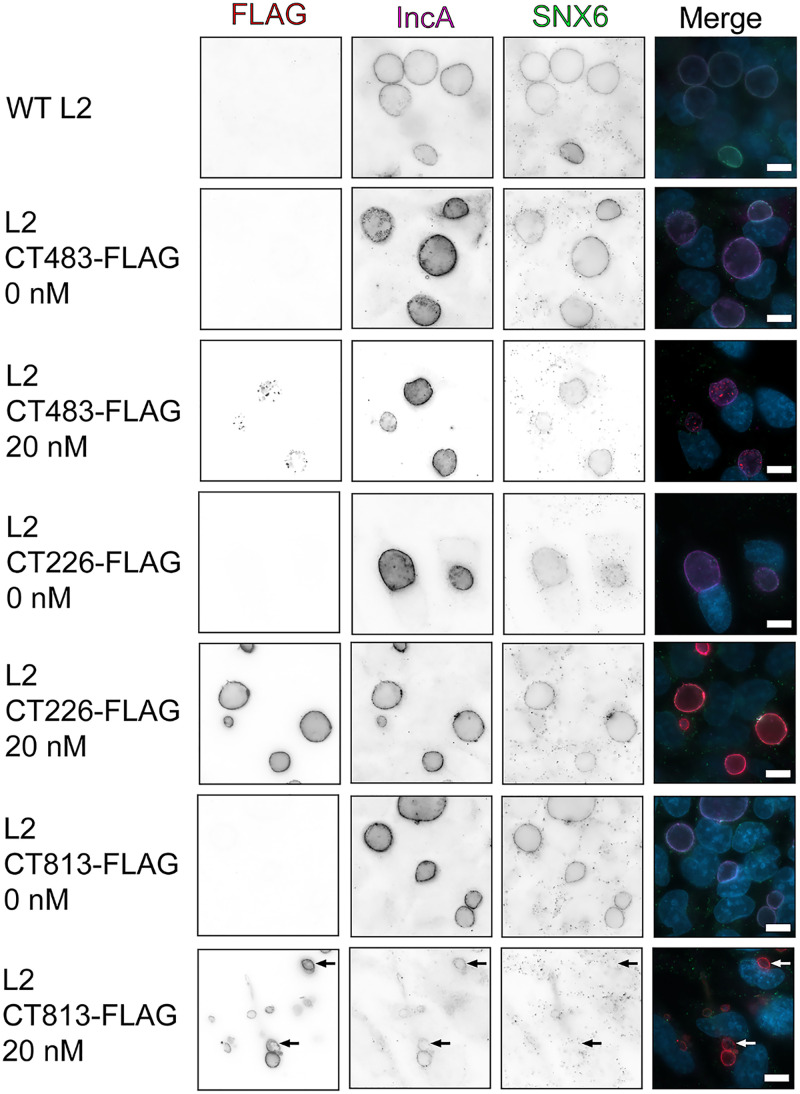
The overexpression of CT813-FLAG from C. trachomatis L2 results in reduced localization of endogenous SNX6 to the inclusion membrane. HeLa cells infected with C. trachomatis L2 transformed strains or wild-type L2 were induced at 7 hpi with 5 or 20 nM aTc or were not induced. Coverslips were methanol fixed at 30 hpi and stained for immunofluorescence to observe expression of the Inc-FLAG constructs (FLAG; red), SNX6 (green), IncA (pink), or DNA (DAPI; blue). Coverslips were imaged at ×63 magnification using the same exposure (scale bar, 10 μm). Arrows indicate C. trachomatis L2 CT813-FLAG inclusions that do not have SNX6. These data are representative of three independent experiments.

Sorting nexin-6 (SNX6) is a eukaryotic protein binding partner of IncE (36, 48). To determine if the loss of IncE in the inclusion membrane due to overexpression of CT813-FLAG also resulted in the loss of detectable SNX6 at the inclusion membrane, C. trachomatis L2 transformed strains were induced for expression or not at 7 hpi, and at 30 hpi, endogenous SNX6 localization was evaluated by indirect immunofluorescence (Fig. 4). Images were captured using the same exposure time for SNX6 to compare the different C. trachomatis L2 strains and induction conditions used. SNX6 localization at the inclusion membrane was not impacted by the overexpression of CT483-FLAG or CT226-FLAG from the C. trachomatis L2 transformed strains (Fig. 4). When CT813-FLAG was overexpressed with 20 nM aTc, the localization of SNX6 at the inclusion membrane was decreased (Fig. 4, arrows), which is consistent with the loss of IncE in the inclusion membrane. However, some inclusions did demonstrate weak SNX6 staining at the inclusion membrane that also had CT813-FLAG staining (Fig. 4). We also evaluated the localization of another eukaryotic protein that is recruited to the inclusion membrane, ceramide transfer protein (CERT) (11, 39). No impact on CERT localization was observed when any of the C. trachomatis L2 transformed strains were induced for expression (Fig. S7). These data indicate that not all eukaryotic proteins that are recruited by Incs may be negatively impacted by the overexpression of a given Inc. However, the overexpression of CT813-FLAG reduced the recruitment of at least one eukaryotic protein, SNX6, the eukaryotic protein binding partner of IncE, to the inclusion membrane.

### Altered developmental cycle progression upon the overexpression of CT813-FLAG from C. trachomatis L2.

Both Western blot and indirect immunofluorescence assays are subject to issues related to the limit of detection. To avoid these limitations, the transcription of *incE*, *incG*, *incA*, and *ct223* was also evaluated using quantitative PCR. HeLa cells were infected with C. trachomatis L2 CT813-FLAG, CT226-FLAG, or CT483-FLAG transformed strains, or with wild-type C. trachomatis L2, and induced or not at 7 hpi with either 5 or 20 nM aTc. At 7, 16, 24, and 36 hpi, RNA and DNA were collected and purified (see Materials and Methods). Transcript data were normalized to genomic DNA (ng cDNA/gDNA) to account for differences in the number of organisms (49).

**FIG 5:**
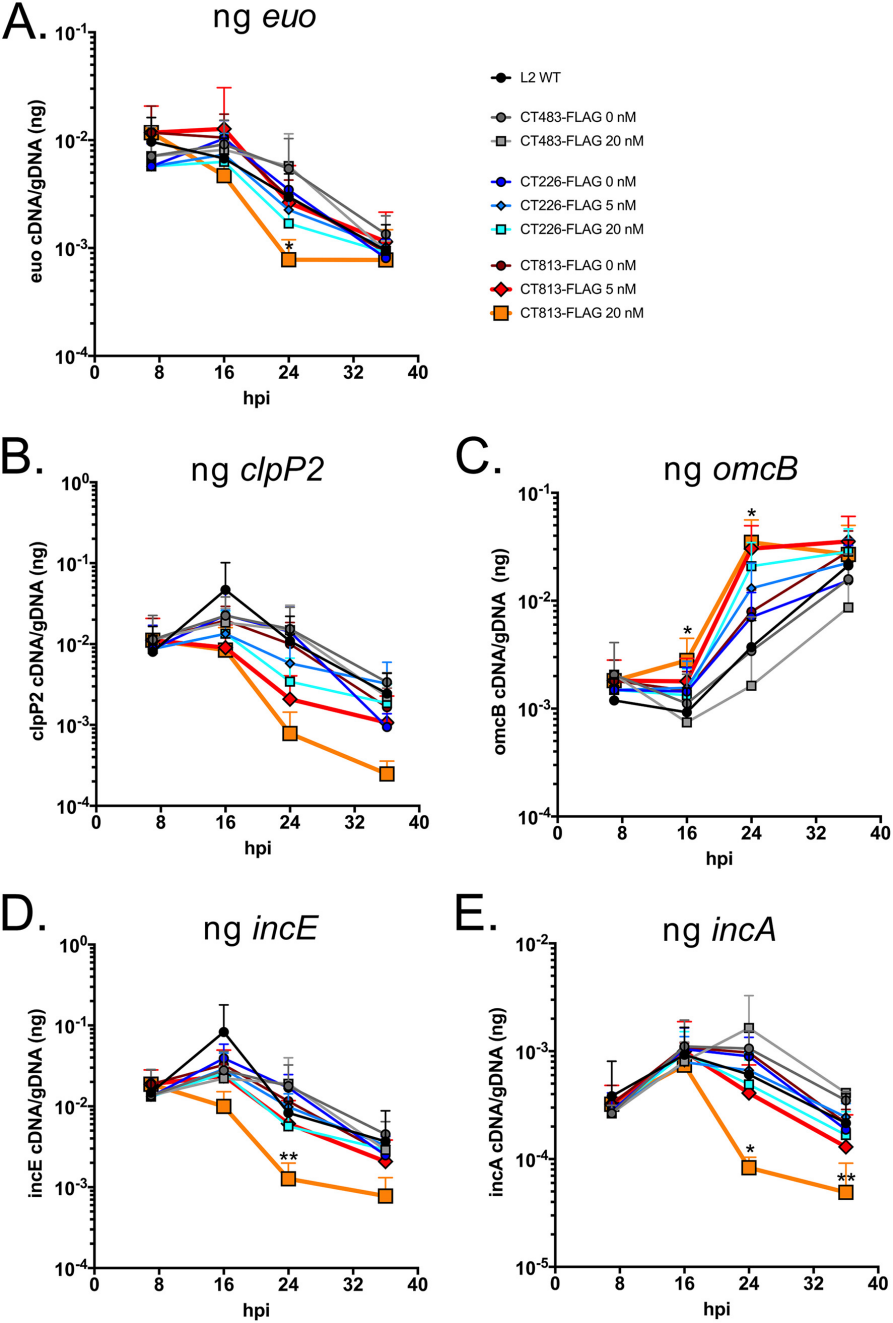
The overexpression of CT813-FLAG from C. trachomatis L2 results in reduced transcription of early and midcycle genes. HeLa cells were infected with C. trachomatis L2 CT813-FLAG, CT226-FLAG, or CT483-FLAG transformed strains, or wild-type C. trachomatis L2, and either not induced or induced at 7 hpi (5 nM or 20 nM aTc). RNA and DNA collected from separate wells of a 6-well plate at 7, 16, 24, and 36 hpi were processed as described in Materials and Methods. Normalized RNA was reverse transcribed to cDNA, and *inc* expression was measured by quantitative PCR. cDNA (ng) was normalized to genomic DNA (ng) for three biological replicates (except for *clpP2*, *n* = 2), and then these data were plotted using GraphPad Prism 8.4.0. Transcript profiles of early gene *euo* (A), midcycle gene *clpP2* (B), late gene *omcB* (C), an early expressed *inc* gene, *incE* (D), and a midcycle *inc* gene, *incA* (E), are shown. Normalized transcripts from wild-type and C. trachomatis L2 CT813-FLAG were log transformed and then analyzed for statistical significance using a paired two-tailed Student's *t* test. Samples that were statistically analyzed were wild type and CT813 at 16 hpi (for *omcB* only), 24 hpi (all genes), and 36 hpi (all genes). Asterisks denote statistical significance: **, *P* ≤ 0.01; *, *P* ≤ 0.05.

First, the impact of the overexpression of Incs on the transcription of *euo*, the prototypical early gene, *clpP2* as a midcycle gene, and *omcB*, a late gene, were examined as a proxy for developmental cycle progression (25, 26). At 24 hpi, transcription of *euo* was significantly decreased for C. trachomatis L2 CT813-FLAG induced with 20 nM aTc compared to wild-type C. trachomatis L2 or strains overexpressing CT226-FLAG or CT483-FLAG (Fig. 5A). The overexpression of CT813-FLAG from C. trachomatis L2 with 20 nM aTc resulted in decreased transcription of midcycle gene *clpP2* earlier in the developmental cycle, but this change in transcript levels was not statistically significant (Fig. 5B). Interestingly, increased transcription of a late gene, *omcB*, was observed when both CT813-FLAG and CT226-FLAG were overexpressed from C. trachomatis L2 transformed strains compared to transcription of *omcB* observed upon aTc addition to control strains, *C. trachomatis* L2 CT483-FLAG and wild-type *C. trachomatis* L2 (Fig. 5C). At both 16 and 24 hpi, transcription of *omcB* was significantly increased for C. trachomatis L2 CT813-FLAG induced with 20 nM aTc compared to wild-type C. trachomatis L2 (Fig. 5C). The altered transcriptional profile observed with 20 nM aTc for C. trachomatis L2 CT813-FLAG was similar to wild-type transcription levels when the induction conditions were reduced to 5 nM aTc for CT813-FLAG (red line, 5 nM aTc) (Fig. 5).

To better characterize the altered expression of Incs on the inclusion membrane, the transcriptional profiles of early expressed *inc* genes, *incE* and *incG*, and midcycle expressed *inc* genes, *incA* and *ct223*, were analyzed. The transcription profile of each *inc* gene analyzed was not altered when CT483-FLAG or CT226-FLAG was overexpressed from C. trachomatis L2 transformed strains compared to the wild-type strain (Fig. 5; Fig. S8). However, the overexpression of CT813-FLAG from C. trachomatis L2 resulted in a significant decrease in the transcription of *incE* at 24 hpi (Fig. 5D) and a significant decrease in *incG* transcript levels at 24 and 36 hpi compared to wild-type C. trachomatis L2 (Fig. S8A; orange line, 20 nM aTc). At 24 and 36 hpi, there was also a statistically significant decrease in the transcription of midcycle *inc* genes, *incA* (Fig. 5E) and *ct223* (Fig. S8B), for C. trachomatis L2 CT813-FLAG induced with 20 nM aTc compared to wild-type C. trachomatis L2. These data suggest that high levels of expression of CT813-FLAG, but not CT226-FLAG, alter the normal developmental cycle progression of C. trachomatis L2.

Given the increase in *omcB* transcript levels, we were interested in understanding if there was also an increase in another late gene, *hctB*. For these studies, we examined *hctB* transcript levels by quantitative PCR (Fig. S9A) and HctB protein levels by immunofluorescence (Fig. S9B). At 7 hpi (the time of addition of aTc), *hctB* transcript levels are elevated in chlamydial strains transformed with CT226-FLAG or CT813-FLAG, regardless of aTc addition and compared to wild-type C. trachomatis L2 (nontransformed) (Fig. S9A). However, the *hctB* transcripts are detected at similar levels for each chlamydial strain at 16 hpi. At 24 hpi, compared to wild-type C. trachomatis, the samples demonstrating the highest *hctB* transcripts were the chlamydial strains induced to express CT226-FLAG (*, *P* = 0.0357) or CT813-FLAG (*, *P* = 0.0273) with 20 nM aTc and CT813-FLAG with 5 nM aTc, consistent with *omcB* expression levels. By 36 hpi, all samples exhibited transcript levels similar to those of nontransformed, wild-type C. trachomatis. To assess HctB protein levels, HeLa cells infected with the indicated strains were induced (or not) with 20 nM aTc at 7 hpi and fixed at 36 hpi, and indirect immunofluorescence was used to detect HctB, construct expression, and chlamydial organisms (Fig. S9B). By indirect immunofluorescence assay, there was no observable increase in HctB in samples infected with the CT226-FLAG or CT813-FLAG transformed strains that were treated with 20 nM aTc compared to wild-type C. trachomatis L2 or strains that were not induced (Fig. S9B). Overall, these results are consistent with the interpretation that overexpression of CT813 alters the chlamydial developmental cycle progression.

### Ultrastructural analysis of C. trachomatis L2 transformed strains after the overexpression of Inc-FLAG constructs in infected HeLa cells.

**FIG 6:**
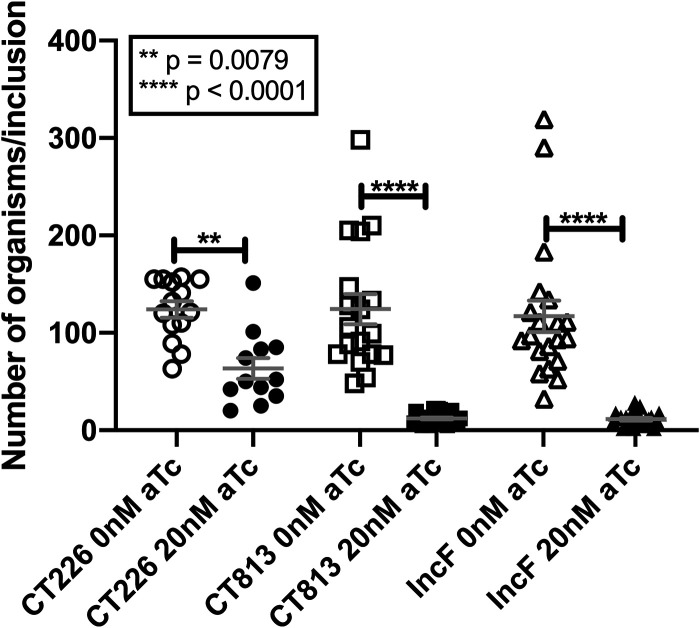
The overexpression of CT813 and IncF from C. trachomatis L2 results in fewer organisms per inclusion. HeLa cells were infected with C. trachomatis L2, CT226-FLAG, CT813-FLAG, or IncF-FLAG transformed strains and either not induced or induced at 7 hpi (5 nM or 20 nM aTc). At 36 hpi, cells were fixed and processed for transmission electron microscopy as previously described (68). The graphed results are the mean with standard error of the mean and are combined data from two biological replicates with the following total numbers of inclusions analyzed: 14 for CT226–0 nM aTc, 12 for CT226–20 nM aTc, 18 for CT813–0 nM aTc, 27 for CT813–20 nM aTc, 20 for IncF–0 nM aTc, and 23 for IncF–20 nM aTc. The data were statistically analyzed in GraphPad Prism (version 8.4.3) using an ordinary one-way ANOVA with Tukey’s multiple-comparison test.

The overexpression of CT813-FLAG from C. trachomatis L2 resulted in a statistically significant increase in late gene transcripts at 16 and 24 hpi compared to wild-type C. trachomatis L2, suggesting that these organisms progress through the developmental cycle more quickly. Ultrastructural analyses were performed to observe the morphological forms (i.e., EBs and RBs) within the inclusion after overexpression of CT813-FLAG, IncF-FLAG, or CT226-FLAG from C. trachomatis L2 transformed strains. Samples were prepared for electron microscopy as described in Materials and Methods from uninduced and induced samples at 36 hpi. The morphological forms observed for the chlamydial strain overexpressing CT483-FLAG were not quantified in the ultrastructural analyses, because CT483 is a membrane protein in the chlamydial cell wall and overexpression of this protein resulted in slightly enlarged bacteria (Fig. S10A). Thus, it is not an appropriate control in regard to the comparison of morphological forms upon the overexpression of Incs, as enumeration of these morphological forms would likely measure an effect of interference with the chlamydial cell wall rather than the impact of Inc overexpression on the development and differentiation of RBs to EBs during the developmental cycle. Further, the enlarged developmental forms caused by overexpression of CT483-FLAG appear to be independent of other metrics of inclusion development used in this study (i.e., inclusion area or infectious progeny) (Fig. 1) or the expression and organization of Incs in the inclusion membrane (Fig. 2 and 4). Consistent with the reduced inclusion area (Fig. 1A), the overexpression of CT813-FLAG (12.19 ± 1.1 organisms per inclusion) or IncF-FLAG (11.39 ± 1.43 organisms per inclusion) from C. trachomatis L2 resulted in smaller inclusions with a statistically significant (*P* < 0.001) decrease in chlamydiae compared to uninduced samples (124.3 ± 15.62 and 117 ± 16.26 organisms per inclusion, respectively) (Fig. 6; see representative transmission electron microscopy [TEM] images in Fig. S10B). There was also a decrease in the number of organisms in inclusions when CT226-FLAG was overexpressed (63.5 ± 10.79 organisms per inclusion) compared to uninduced samples (124 ± 8.3 organisms per inclusion), and these differences were also statistically significant (*P* = 0.0079). We then quantified the morphological forms (EB, intermediate body [IB], or RB) observed within each inclusion. Because intermediate bodies are those organisms that are in the process of secondary differentiation from an RB to an EB, we combined their numbers with the EBs. Specifically, if organisms were further advanced within the developmental cycle, then there should be more EBs and IBs than RBs than under control conditions. While enumerating EBs, IBs, and RBs, we also encountered organisms that we labeled “indeterminant” because they did not morphologically resemble well-defined developmental forms (Fig. S10C). While the overall number of detectable bacteria was decreased when CT813-FLAG or IncF-FLAG was induced for expression with 20 nM aTc, the percentage of EBs and IBs was not statistically different from that of uninduced samples (Fig. S11A). However, there were fewer RBs per inclusion upon overexpression of CT813, with 25.23% ± 3.9% compared to 40.90% ± 3.1% in uninduced samples (*P* = 0.0461) (Fig. S11B). Notably, in all samples, overexpression of any Inc increased the incidence of organisms with indeterminant morphologies, which was most pronounced under the CT813-FLAG overexpression conditions (Fig. S11C). Interestingly, these data indicate that chlamydiae that are overexpressing CT813-FLAG produce fewer overall organisms, and fewer RBs indicate that secondary differentiation may be occurring at a different rate than in control samples. In contrast, chlamydiae that are overexpressing IncF-FLAG seem to produce fewer overall organisms, but the developmental cycle likely progresses normally.

## DISCUSSION

**FIG 7:**
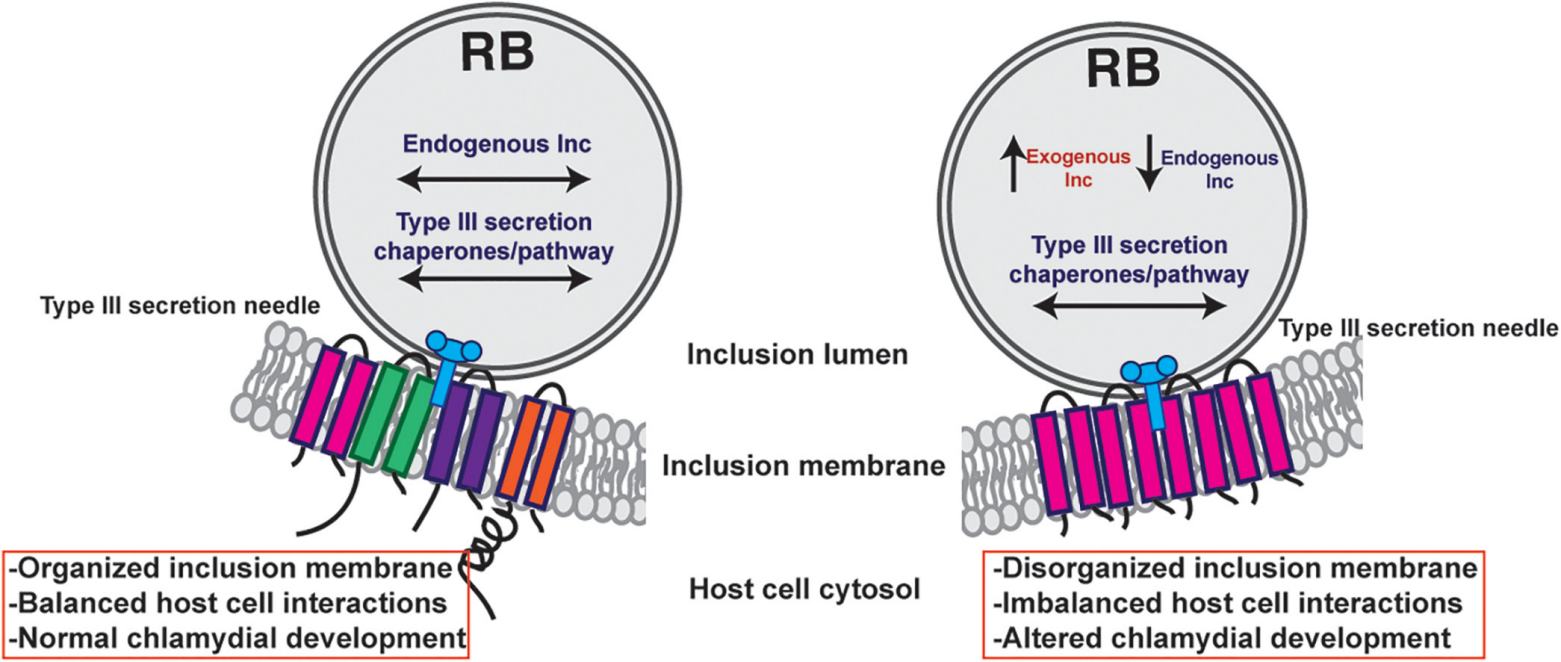
Model of the consequences of *inc* overexpression. In wild-type Chlamydia, *inc* expression is temporal, which results in an inclusion membrane that can expand with the growing number of organisms and optimal host-Chlamydia interactions that maximizes the amount of infectious progeny produced. When certain Incs, like IncF and CT813, are produced at high amounts, they are type III secreted and inserted in the inclusion membrane. It is unknown at this point if these exogenous Incs “flood” the type III secretion system at the expense of endogenous Incs, which causes a decrease in endogenous *inc* transcripts. The ultimate result is a disorganized inclusion membrane that has altered interactions with the host cell (e.g., loss of SNX6 recruitment) and, thus, altered chlamydial development. These data indicate that inclusion membrane dynamics, which includes inclusion membrane organization and the resulting Inc-host interactions, contributes to optimal chlamydial development.

The impact of the overexpression of various Incs was evaluated to test our hypothesis that some Incs function in the structure and organization of the inclusion membrane. Collectively, our data demonstrate that CT813 and IncF likely function in this capacity. The overexpression of these Incs altered the localization of endogenous Incs in the inclusion membrane, which may contribute to the observed negative impacts on inclusion biogenesis and chlamydial development (Fig. 1; see Fig. S1 in the supplemental material). Furthermore, the abundance of endogenous Incs (i.e., IncE, IncG, IncA, and CT223) in the inclusion membrane was reduced by the overexpression of CT813-FLAG or IncF-FLAG (Fig. 2 and 3; Fig. S2 to S6). Importantly, disrupted IncE in the inclusion membrane also reduced the recruitment of its eukaryotic protein binding partner, SNX6, to the inclusion (Fig. 4). Upon CT813-FLAG overexpression, we measured changes in developmental cycle progression, which indicated an earlier increase in transcripts of the late gene *omcB* concomitant with earlier decreases in early and midcycle gene transcripts (Fig. 5; Fig. S8). We also measured by electron microscopy a quantifiable decrease in RBs (Fig. 6; Fig. S10 and S11). In support of our hypothesis that certain Incs are involved in the structure of the inclusion membrane, not all of the Incs (e.g., CT226-FLAG) that were overexpressed negatively impacted inclusion development (Fig. 1 and 3). From these data, we propose a model of how this may impact inclusion development (Fig. 7).

To date, many studies have utilized C. trachomatis L2 Inc overexpression and knockout strains to validate a specific Inc protein and eukaryotic protein interaction (50–52). As antibodies against Incs are not widely available, these studies rarely use endogenous Incs as an indicator of inclusion membrane development. As such, it is difficult to compare our findings with those previously published. Instead, inclusion diameter is frequently used as an indicator of chlamydial development. For example, Dickinson et al. measured the inclusion diameter at 24 hpi for various C. trachomatis L2 Inc-APEX2 transformed strains that were induced for construct expression at the time of infection (1 ng/ml, ∼ 2.15 nM aTc) (53). Under these conditions, the reported inclusion diameter was decreased for C. trachomatis L2 IncB-APEX2, CT223-APEX2, CT813(InaC)-APEX2, IncC-APEX2, and IncA-APEX2 transformed strains (53). Of interest, under these overexpression conditions, IncB-APEX2 was observed uniformly around the inclusion rather than in microdomains (46, 54), but the presence of IncA and CT223 at the inclusion membrane was not impacted (53), which may indicate that the localization of some but not all Incs can impact the localization of other Incs in the inclusion membrane. Others in the field may have noticed a negative impact of the overexpression of certain Incs on inclusion expansion but did not report this. For example, the interaction of VAPA and VAPB with CT005 (IncV) was performed using a C. trachomatis L2 CT005-FLAG strain induced for expression over short 4-h incubation with higher levels of aTc (20 ng/ml aTc or ∼43.2 nM aTc) (51). We have observed reduced inclusion size upon extended periods of CT005 overexpression (data not shown). Similarly, certain C. trachomatis L2 *inc* knockout strains also displayed altered inclusion size while others did not. A study of C. trachomatis L2 *CT229*::*bla*, *incC*::*bla*, *CT383*::*bla*, and *CT449*::*bla* strains resulted in smaller, nonfusing, inclusions (15, 55, 56) but no observable differences in the intensity of IncA (15). In contrast, other mutant C. trachomatis L2 strains, including those lacking *ct288* (15, 57) or *ct228*, exhibited no growth defects (34). Together, these data indicate that certain Incs are likely involved in the structural integrity of the inclusion membrane whereas others are dispensable for this function. Interestingly, both the overabundance and the loss of the same Inc can negatively impact normal inclusion expansion. Thus, not surprisingly, the careful coordination of inclusion membrane composition is important for optimal chlamydial development.

The observation of altered inclusion membrane composition corresponded with altered developmental cycle progression measured by reverse transcription quantitative PCR (RT-qPCR). The altered transcriptional profile by the overexpression of CT813-FLAG was observed for genes encoding both type III secreted effectors and non-type III secreted effectors. This suggests that the loss of endogenous Incs, in this scenario, is not related to overloading the type III secretion or chaperone availability (Fig. 5; Fig. S6). In support, the induction of CT813-FLAG later in the developmental cycle also negatively impacted the localization of Incs that are expressed early in the developmental cycle (i.e., IncE and IncG) (Fig. S6). Others have overexpressed non-type III secretion substrates (e.g., wild-type ClpP1, ClpP2, and ClpX) with no impact on inclusion size or progeny production (44, 58). These data support the findings that the overexpression of certain Incs results in the misorganization of Incs in the inclusion membrane that negatively impacts development. In addition, Weber et al. overexpressed various Incs and putative Incs that did not demonstrably impact inclusion size (23), which indicates that only the overexpression of certain chlamydial proteins results in reduced inclusion area. Why overexpression of CT813-FLAG might lead to an altered progression through the developmental cycle and what this means for cues that drive this process are not known at this time. Further work is required to determine if this is an effect specific to CT813 or occurs when other Incs (not assessed here) are overexpressed.

Of interest, the overexpression of either IncF-FLAG or CT813-FLAG resulted in smaller inclusions and altered Inc composition; yet, by bacterial two-hybrid analyses, CT813 exhibited only homotypic interactions whereas IncF exhibited homotypic and heterotypic interactions with numerous other Incs (28, 43). It is possible that during infection, CT813 is capable of interacting with other Incs. In addition, Inc-Inc interactions may occur primarily through transmembrane domain regions within the inclusion membrane itself (28), while the C-terminal regions exposed to the host cytosol may preferentially bind eukaryotic proteins. Alternatively, there may be dual functions for some Inc proteins in inclusion stability and/or expansion through the binding of eukaryotic proteins, since CT813-FLAG has been reported to bind the eukaryotic proteins Arf1 and Arf4 (50). However, neither knockdown or overexpression of Arf1 or Arf4 resulted in an observable change in inclusion size (50). Similarly, we did not observe an impact on Golgi fragmentation into ministacks around the inclusion when CT813-FLAG was overexpressed (data not shown). Our observation of smaller inclusions may also be the result of the different functions of two different Incs, whereby CT813 binds eukaryotic proteins necessary for inclusion expansion. In contrast, IncF has recently been shown to transiently bind to the eukaryotic SNARE protein VAMP3, which also binds to IncG and CT813 (41). It is unclear what the function of these Inc-VAMP3 interactions is, but notably, siRNA knockdown of VAMP3 results in smaller inclusions (41). Thus, the overexpression of either IncF or CT813 negatively impacted inclusion expansion either by disorganizing the content of the inclusion membrane and/or by altering Inc-host interactions that contribute to inclusion expansion.

The overexpression of CT813-FLAG impacted the recruitment of at least one eukaryotic protein (i.e., SNX6) that is normally recruited to the inclusion membrane during C. trachomatis infection. If the overexpression of a specific Inc results in increased (or decreased) localization of a eukaryotic protein at the inclusion, then we cannot exclude the possibility that this outcome is the result of the loss of an Inc in the inclusion membrane that could function to inhibit a specific host protein at the inclusion membrane. For example, IncA has been reported to inhibit VAMP8 localization at the inclusion (59). Concomitantly, data in our laboratories indicate that there is increased VAMP3 localization at the inclusion membrane of a C. trachomatis L2 *incA* knockout (41). Furthermore, the current study indicates that it is possible that the differences in eukaryotic proteins identified in proximity labeling studies to map protein-protein interaction at the inclusion membrane might be a result of changes in inclusion membrane composition (43, 53, 60).

This study analyzed the impact of the overexpression of specific Incs on inclusion composition and expansion to better understand the function of Incs. There are no previously published reports of the negative impact of Inc overexpression on the composition of endogenous Incs in the inclusion membrane. In future studies, it will be important to evaluate the composition of Incs in inclusions formed by both C. trachomatis L2 *inc* knockout and overexpression strains. This can be achieved by monitoring the composition of endogenous Incs using immunofluorescence assays as a way to ensure normal C. trachomatis development. This work also highlights the significance of maintaining an appropriate expression level of Incs for studies that aim to capture the chlamydial and eukaryotic protein-protein interactions at the inclusion membrane. The use of either short induction times for overexpression studies or allelic exchange to tag a targeted *inc* could also be used to avoid the negative impact of high expression levels of certain Incs. Overall, these data suggest that there is a specific composition of Incs in the inclusion membrane that is required for optimal inclusion biogenesis and C. trachomatis development (Fig. 7).

## MATERIALS AND METHODS

### Antibodies and reagents.

Primary antibodies used included the following: mouse anti-FLAG (Sigma), rabbit anti-FLAG (Sigma), mouse anti-GAPDH (glyceraldehyde-3-phosphate dehydrogenase) (EMD Millipore), mouse anti-sorting nexin-6 (SNX6) clone D-5 (Santa Cruz Biotechnology; sc-365965), goat anti-MOMP (Meridian, Memphis, TN), guinea pig anti-L2, sheep anti-IncA (made to order by Seramum Diagnostica GmbH, Heidesee, Germany), rabbit anti-IncE, rabbit anti-IncG, rabbit anti-CT813, and rabbit anti-HctB (kind gifts from T. Hackstadt, NIAID, Rocky Mountain Laboratories, Hamilton, MT), and mouse anti-CT223 (kind gift from R. Suchland, University of Washington, WA, and D. Rockey, Oregon State University, OR). Secondary antibodies used for immunofluorescence were donkey anti-mouse-, rabbit-, or sheep-647, donkey anti-mouse- or rabbit-594, donkey anti-mouse- or rabbit-488, and donkey anti-mouse-, guinea pig-, or rabbit-405 (Jackson ImmunoLabs and Invitrogen). DAPI (4′,6-diamidino-2-phenylindole) was used to visualize DNA as indicated. For Western blots, after incubation with the indicated primary antibody followed by the appropriate secondary antibodies conjugated to IRDye 680LT or IRDye 800 CW (LiCor Biosciences, Lincoln, NE), membranes were imaged using Azure c600 (Azure Biosystems, Dublin, CA) and processed using Adobe Photoshop Creative Cloud (Adobe).

### Organisms and cell culture.

HeLa 229 cells (American Type Culture Collection [ATCC]; Manassas, VA; CCL-2.1) were cultured at 37°C with 5% CO_2_ in biotin-free Dulbecco’s modified Eagle’s medium (DMEM) (Gibco; Grand Island, NY) that was supplemented with 10% heat-inactivated fetal bovine serum (FBS; HyClone, Logan, UT) for routine tissue culture with 10 μg/ml gentamicin (Gibco-BRL/Life Technologies; Grand Island, NY). HeLa cells were used to propagate Chlamydia trachomatis serovar L2 (LGV 434) for purification by using established protocols (61, 62). Chlamydial titers were determined using conventional protocols to establish multiplicities of infection (MOI) based on inclusion-forming units (IFU) and determined in HeLa cells as previously described (62, 63). McCoy cells (ATCC, Manassas, VA; CRL-1696) were cultured at 37°C with 5% CO_2_ in biotin-free DMEM (Gibco, Grand Island, NY) that was supplemented with 10% FBS (HyClone, Logan, UT) used for C. trachomatis L2 (LGV 434) transformation experiments. HeLa cells, McCoy cells, and density gradient-purified C. trachomatis strains are routinely tested for *Mycoplasma* spp. (LookOut mycoplasma PCR detection kit; Sigma; St. Louis, MO).

### Creation of Inc fusion constructs and transformation into C. trachomatis L2.

Competent Escherichia coli 10-β cells were used in cloning projects. C. trachomatis serovar D naming conventions are used in this article, although *inc* genes were originally amplified from C. trachomatis L2 434/Bu genomic DNA. For clarity, the C. trachomatis serovar L2 434/Bu gene names are included in parentheses next to the appropriate constructs. For the construction of pBOMB4-Tet-IncF-FLAG (*CTL0372*), IncF-FLAG was amplified from pTLR2-IncF-APEX2 (primers are listed in Table S1 in the supplemental material) (40). For the construction of pBOMB4-Tet-CT813-FLAG, CT813 with the C-terminal FLAG tag was amplified from a lab-generated pTLR2-CT813-APEX2 construct in which *CTL0184* was amplified from C. trachomatis serovar L2 genomic DNA (Table S1). The untagged CT813 strain was generated from genomic DNA using the primers in Table S1. The PCR products were cloned and inserted into the mCherry site of pBOMB4-Tet (flanked by EagI/KpnI cut sites) (a gift from T. Hackstadt, NIAID, Rocky Mountain Laboratories, Hamilton, MT) using the NEBuilder hifi assembly cloning kit (NEBuilder). The final constructs were transformed into methyltransferase-deficient *E. coli* (*dam*^−^, *dcm*^−^). All constructs were confirmed by sequencing (Eurofins MWG Operon, Huntsville, AL) and restriction digestion. C. trachomatis L2 transformations were performed as previously described (64, 65). pBOMB4-Tet-CT813-FLAG and pBOMB4-Tet-IncF-FLAG were transformed in the presence of 1 U/ml penicillin and 1 μg/ml cycloheximide. Stable clones were plaque purified (64, 66) and subsequently density gradient purified. C. trachomatis L2 pBOMB4-Tet-CT483-FLAG (*CTL0744*) was a kind gift from M. Weber (University of Iowa, Iowa City, IA) (23), and the C. trachomatis L2 pBOMB4-Tet-CT226-FLAG (*CTL0478*) strain (43) and C. trachomatis L2 pBOMB4-mCherry-GFP strain were described previously (44).

HeLa cells were infected by centrifugation (using an MOI of 0.75 unless otherwise indicated) using wild-type C. trachomatis L2 or L2 transformed with a plasmid that expresses IncF-FLAG, CT813-FLAG, CT483-FLAG, or CT226-FLAG. C. trachomatis L2 IncF-FLAG and C. trachomatis L2 CT226-FLAG were infected in DMEM plus 10% FBS containing 1 U/ml penicillin and 1 μg/ml cycloheximide. C. trachomatis L2 CT483-FLAG-infected wells contained DMEM plus 10% FBS with 0.1 U/ml penicillin and 1 μg/ml cycloheximide. Wild-type C. trachomatis L2 was infected in DMEM plus 10% FBS containing 1 μg/ml cycloheximide.

### C. trachomatis L2 infection of HeLa cells and inclusion area measurements.

HeLa cells seeded at 1.0 × 10^5^ cells/well in a 24-well plate containing a glass coverslip were infected with C. trachomatis L2 CT813-FLAG, IncF-FLAG, CT226-FLAG, or CT483-FLAG transformed strains or wild-type C. trachomatis L2. At 7 hpi, the C. trachomatis L2 strains were induced with 1 (C. trachomatis L2 IncF-FLAG only), 5, or 20 nM aTc. As a control, wild-type C. trachomatis L2 was treated, or not, with 20 nM aTc. Coverslips were methanol fixed at 36 hpi and stained for immunofluorescence to determine inclusion area. A minimum of 100 inclusions per condition were measured using ImageJ. The inclusion area (μm^2^) and standard deviation were plotted using GraphPad Prism 8.4.0 for three biological replicates (Fig. 1A and Fig. S1A). These data were analyzed for statistical significance using a one-way analysis of variance (ANOVA) and Tukey’s multiple-comparison test.

Additional controls including C. trachomatis L2 transformed with CT813 (no epitope tag) and C. trachomatis L2 transformed with pBOMB-mCherry, a vector that constitutively expresses mCherry (i.e., empty vector control) (44), were also used. For these experiments, HeLa cells seeded on coverslips were infected with C. trachomatis L2 CT813 and induced, or not, at 7 hpi with 20 nM aTc. Coverslips were methanol fixed at 36 hpi and stained for immunofluorescence to observe construct expression (anti-CT813 antibody; red), IncA (green), or DNA (DAPI; blue) (Fig. S1B). For the empty vector control, C. trachomatis-infected HeLa cells were fixed at 24 hpi using 3.25% formaldehyde and 0.025% glutaraldehyde to preserve the mCherry and stained for immunofluorescence to observe IncA (green) or DNA (DAPI; blue). Coverslips were imaged at ×63 magnification.

### Enumeration of infectious progeny and plasmid retention experiments.

HeLa cells infected in duplicate with C. trachomatis L2 CT813-FLAG, IncF-FLAG, CT226-FLAG, or CT483-FLAG transformed strains, or wild-type C. trachomatis L2, were induced for expression at 7 hpi using 1 nM, 5 nM, or 20 nM aTc. At 36 hpi, infected monolayers were lysed by vortexing with glass beads, serial dilution, and infection onto a fresh monolayer of HeLa cells (i.e., for secondary infection) on glass coverslips. The secondary infection was performed with medium containing penicillin to enumerate infectious progeny that retained the pBOMB4 plasmid. Infectious progeny were enumerated and reported as inclusion-forming units (IFU)/ml. The number of infectious progeny (IFU/ml) and standard deviation were plotted using GraphPad Prism 8.4.0 for three biological replicates. These data were analyzed for statistical significance using a one-way ANOVA and Tukey’s multiple-comparison test. Only inclusions with the wild-type phenotype were enumerated for the infectious progeny assay. Plasmid loss was indicated by inclusions containing aberrant bacteria when grown in the presence of medium containing penicillin (i.e., sensitivity due to the loss of the plasmid-borne *bla* resistance gene). For the plasmid loss experiments, glass coverslips were stained using anti-MOMP antibodies and visualized at ×63 magnification to detect enlarged bacteria within the inclusion (consistent with the morphology of aberrant bacteria). To enumerate the percentage of inclusions containing aberrant bacteria, the number of inclusions that contained aberrant bacteria (inclusion criteria included one or more aberrant bacteria within the inclusion) were divided by the total number of inclusions counted. The percentage of aberrant bacteria and the standard deviation were plotted using GraphPad Prism 8.4.0 for three biological replicates. Raw progeny data (Fig. 1B) for C. trachomatis L2 strains were as follows (the nanomolar concentrations are for aTc): wild-type L2, 4.64 × 10^8^ ± 8.47 × 10^7^ IFU/ml; CT483-FLAG 0 nM, 8.16 × 10^7^ ± 4.23 × 10^7^ IFU/ml; CT483-FLAG 20 nM, 6.70 × 10^7^ ± 1.11 × 10^7^ IFU/ml; CT226-FLAG 0 nM, 2.36 × 10^8^ ± 2.40 × 10^8^ IFU/ml; CT226-FLAG 5 nM, 1.51 × 10^8^ ± 1.46 × 10^8^ IFU/ml; CT226-FLAG 20 nM, 5.10 × 10^7^ ± 6.24 × 10^7^ IFU/ml; CT813-FLAG 0 nM, 1.52 × 10^7^ ± 1.57 × 10^7^ IFU/ml; CT813-FLAG 5 nM, 2.19 × 10^6^ ± 9.43 × 10^5^ IFU/ml; and CT813-FLAG 20 nM, 1.94 × 10^5^ ± 2.65 × 10^5^ IFU/ml.

### Detection of C. trachomatis proteins by Western blotting.

HeLa cells seeded at 1.0 × 10^6^ cells/well in a 6-well plate were infected with C. trachomatis L2 CT813-FLAG, CT226-FLAG, or CT483-FLAG transformed strains or wild-type C. trachomatis L2 and either not induced or induced at 7 hpi (5 nM or 20 nM aTc). At 36 hpi, infected monolayers were lysed in radioimmunoprecipitation (RIPA) buffer modified with 1% Triton X-100, HALT protease inhibitor with EDTA (Pierce), clasto-lactacystin β-lactone (Cayman Chemical; CAS 154226-60-5), and nuclease (Pierce) (43). Lysates were sonicated and clarified by centrifugation (13,000 rpm for 10 min). At 48 hpi, cell lysates were collected in fresh 8 M urea supplemented with 1% SDS, 10 mM Tris (pH 7.4), 2.5% β-mercaptoethanol, and nuclease. Lysates were quantified using the EZQ protein quantitation kit (Thermo Fisher Scientific). Equal amounts of solubilized lysates were loaded and separated by 12% SDS-PAGE and transferred to polyvinylidene difluoride (PVDF) to blot for C. trachomatis proteins (see “Antibodies and reagents”). Membranes were also blotted against GAPDH, which served as a loading control (Fig. 2).

### Determination of Inc expression and localization by indirect immunofluorescence.

HeLa cells were seeded at 1.0 × 10^5^ cells/well in a 24-well plate containing a glass coverslip. Wells were infected with wild-type C. trachomatis L2 or L2 transformed with a plasmid that encodes IncF-FLAG, CT813-FLAG, CT483-FLAG, or CT226-FLAG. C. trachomatis L2 IncF-FLAG or CT226-FLAG transformants (MOI = 0.75) were infected by centrifugation in DMEM plus 10% FBS containing 1 U/ml penicillin and 1 μg/ml cycloheximide. C. trachomatis L2 CT483-FLAG transformant (MOI = 0.75)-infected wells received 0.1 U/ml penicillin and 1 μg/ml cycloheximide. Wild-type C. trachomatis L2 was infected (MOI = 0.5) in DMEM plus 10% FBS containing 1 μg/ml cycloheximide. At 7 hpi, the C. trachomatis L2 strains were induced with 1 nM aTc for IncF-FLAG or 5 and 20 nM aTc for all other strains and wild-type C. trachomatis L2, respectively. At 36 hpi, glass coverslips were fixed in methanol and then processed for immunofluorescence. Individual coverslips were imaged using the same exposure on a Zeiss Axio Imager.Z2 (without the ApoTome.2) at ×63 magnification. Individual color channels were converted to black and white and then inverted. The intensity of IncE and IncG was quantified using ImageJ. For each image, the background integrated density was subtracted from individual images, and the intensity was normalized to the inclusion perimeter (integrated density/μm). The intensity and standard deviation were plotted using GraphPad Prism 8.4.0. Samples were analyzed for statistical significance using a one-way ANOVA and Tukey’s multiple-comparison test. For the 36-hpi data set, a minimum of 80 inclusions per experiment were measured for each condition for three independent experiments. The 18-hpi data are representative of two independent experiments. For these experiments, HeLa cells were infected with wild-type C. trachomatis L2 and methanol fixed at 18 hpi (Fig. 3B) to assess the expression and localization of IncE and IncG on early inclusions to control for differences in inclusion size. For these experiments, infected cells were stained for IncE or IncG (green) and DNA (DAPI; blue). For the 18-hpi time point only, IncE and IncG were imaged using a shorter exposure time to prevent oversaturation. These data were normalized to the exposure time used for the imaging of 36 hpi inclusions for IncE and IncG, respectively. The mean integrated density/μm is indicated in the red box for each sample measured. **** indicates a *P* of <0.0001 between C. trachomatis L2 transformed strains, and #### indicates a *P* of <0.0001 between different C. trachomatis L2 strains. In some experiments, C. trachomatis L2 CT813-FLAG-infected HeLa cells (Fig. S6) were induced at 14.5 hpi using 5 nM or 20 nM aTc and then methanol fixed at 36 hpi and stained for immunofluorescence to observe construct expression (FLAG; red), IncA (pink), DNA (DAPI; blue), and either IncE (top panel; green) or IncG (bottom panel; green). Coverslips were imaged using a Zeiss Axio Imager.Z2 without the ApoTome.2 at ×63 magnification using the same exposure for each sample.

### Visualization of SNX6 and CERT at the inclusion membrane.

HeLa cells infected with C. trachomatis L2 transformed strains or wild-type L2 were induced, or not, at 7 hpi with 5 or 20 nM aTc. Coverslips were methanol fixed at 30 hpi and stained for immunofluorescence to observe expression of the Inc-FLAG constructs (FLAG; red), sorting nexin-6 (SNX6) (green), IncA (pink), or DNA (DAPI; blue). Arrows indicate C. trachomatis L2 CT813-FLAG inclusions that do not have SNX6 colocalized with the inclusion membrane. Coverslips were imaged using Zeiss Axio Imager.Z2 with ApoTome.2 with ×100 magnification. Individual color channels were converted to black and white and then inverted.

To visualize ceramide transfer protein (CERT) localization, HeLa cells infected with C. trachomatis L2 transformed strains or wild-type L2 were induced at 7 hpi with 5 or 20 nM aTc or were not induced. Coverslips were fixed with 2% paraformaldehyde at 36 hpi, permeabilized with 0.5% Triton X-100, and then stained for immunofluorescence to observe expression of the Inc-FLAG constructs (FLAG; pink), CERT (red), MOMP (green), or DNA (DAPI; blue). Coverslips were imaged using Zeiss Axio Imager.Z2 at ×63 magnification. Individual color channels were converted to black and white and then inverted.

### Nucleic acid extraction and RT-qPCR.

HeLa cells were seeded at 1.0 × 10^6^ cells/well in a 6-well plate with a glass coverslip in each well to confirm construct expression. HeLa cells were infected with C. trachomatis L2 CT813-FLAG, CT226-FLAG, or CT483-FLAG transformed strains, or wild-type C. trachomatis L2, and either not induced or induced at 7 hpi (5 nM or 20 nM aTc). RNA and DNA were collected from separate wells of a 6-well plate at 7, 16, 24, and 36 hpi. Prior to the collection of nucleic acids, coverslips in each well were fixed and stained for immunofluorescence to confirm the expression of the construct. RNA was extracted from infected HeLa cells using TRIzol (Invitrogen) as previously described (67). Briefly, RNA samples were treated with Turbo DNA-free (Ambion/Thermo Fisher) by following the manufacturer’s instructions to remove DNA contamination, and cDNA was synthesized from DNA-free RNA using random nonamers (New England BioLabs, Ipswich, MA) and SuperScript III reverse transcriptase (RT; Invitrogen/Thermo Fisher) per the manufacturer’s instructions. Reaction end products were diluted 10-fold with molecular biology grade water, aliquoted, and stored at −80°C for later use. Equal volumes of each reaction mixture were used in 25-μl quantitative PCR (qPCR) mixtures with SYBR green master mix (Applied Biosystems) and quantified on a QuantStudio 3 (Applied Biosystems/Thermo Fisher) by using the standard amplification cycle with a melting curve analysis. Results were compared to a standard curve generated against purified C. trachomatis L2 genomic DNA or pBOMB4-Tet plasmid DNA when appropriate. DNA was extracted from infected HeLa cells using the DNeasy blood and tissue kit (Qiagen, Hilden, Germany). Equal amounts of DNA were used in qPCR with a *euo* primer set to quantify chlamydial genomes. The transcript data were normalized to genomic DNA. All qPCR primer sequences used in this study are listed in Table S1. Samples were analyzed in technical triplicate for three biological replicates. RT-qPCR results were normalized for efficiency, with typical results demonstrating an *r*^2^ of ≥0.995 and greater than 90% efficiency. cDNA (ng) was normalized to genomic DNA (ng) and plotted using GraphPad Prism 8.4.0. To analyze for statistical significance, normalized samples (ng cDNA/gDNA) for wild-type and L2 CT813-FLAG transformed strains were log transformed and compared using the Student's *t* test.

### Electron microscopy determination of bacterial morphology and localization.

HeLa cells were seeded at 1.0 × 10^6^ cells/well in a 6-well plate. A glass coverslip was included in each well to confirm construct expression by indirect immunofluorescence assay. Individual wells were infected with wild-type C. trachomatis L2 or L2 transformed with a plasmid that encodes IncF-FLAG, CT813-FLAG, CT483-FLAG, or CT226-FLAG. C. trachomatis L2 IncF-FLAG and CT226-FLAG (MOI = 0.75) were infected by centrifugation in DMEM plus 10% FBS containing 1 U/ml penicillin and 1 μg/ml cycloheximide. C. trachomatis L2 CT483 (MOI = 0.75)-infected wells received 0.1 U/ml penicillin and 1 μg/ml cycloheximide. Wild-type C. trachomatis L2 was infected (MOI = 0.5) in DMEM plus 10% FBS containing 1 μg/ml cycloheximide. At 7 hpi, the C. trachomatis L2 strains were induced with 1 nM aTc for IncF-FLAG and 5 and 20 nM aTc for all other strains and wild-type C. trachomatis L2, respectively. At 36 hpi, glass coverslips were fixed in methanol for 6 min and then processed for immunofluorescence confirmation of construct expression as described above.

The wells intended for electron microscopy were prepared using a protocol adapted from a report by Lucas et al. (68). Briefly, infected HeLa cells were detached with trypsin and pelleted by centrifugation at 3,000 × *g* for 5 min at 4°C. Cell pellets were washed with Dulbecco’s phosphate-buffered saline (PBS), resuspended in fixing solution (2% glutaraldehyde, 2% paraformaldehyde in 0.1 M phosphate buffer), and delivered to the University of Nebraska Medical Center electron microscopy core to be processed. In brief, the samples were postfixed with 1% osmium tetroxide, stained with toluidine blue, dehydrated with a series of increasing ethanol concentrations, embedded, and sectioned. Sections were placed on 200 mesh uncoated copper grids (Ted Pella, Inc.), stained with uranyl acetate and Reynold’s lead citrate, and examined using a Tecnai G2 Spirit (FEI) transmission electron microscope (TEM) operated at 80 kV. Representative electron micrographs are shown for two biological replicates.
