# Supplementary material for: Inclusion Membrane Growth and Composition Are Altered by Overexpression of Specific Inclusion Membrane Proteins in Chlamydia trachomatis L2
Source: Infect Immun. 2021 Jun 16;89(7):e00094-21. doi: 10.1128/IAI.00094-21 (PMC8208519; doi:10.1128/IAI.00094-21)
Supplement: Supplemental file 1 — Table S1. Download IAI.00094-21-s0001.pdf, PDF file, 23 KB [file iai.00094-21-s0001.pdf]

Table S1. Primers used in this study		
Primer type	Fwd/Rev	primer sequence
standard cloning primers		
pBOMB4-Tet-IncF-FLAG	F'	gatctaaagaggagaaaggatctgcATGGGAGACGTAATGATACAG
	R'	acatatttgaatggtcgaccggtacTTACTTATCGTCGTCATCCTTG
pBOMB4-Tet-CT813-FLAG	F'	gatctaaagaggagaaaggatctgcATGACTACTCTTCCCAATACTTG
	R'	acatatttgaatggtcgaccggtacttaCTTATCGTCGTCATCCTTGTAG
pBOMB4-Tet-CT813	F'	gatctaaagaggagaaaggatctgcATGACTACTCTTCCCAATACTTG
	R'	tttgaatggtcgaccggtacCTATATCGAACCACGTCTTCCT
qPCR primers		
euo	F'	CGAAGACTACTCGTTGGGAAATA
	R'	AACAGAAGCTCTCCTTGATAAGT
omcB	F'	CGGTAGGATCTCCCTATCCTATT
	R'	CGAACTCTGCTTCACATGGTA
incE	F'	GGTGTTGACGAAAGGAACAAC
	R'	CAGCGATTCCCAAAGCTAAAC
incG	F'	CGAAATGCTTACAAACGGCTAAG
	R'	CAGTACCGCCGCTGATAATATG
incA	F'	TCTGATCGCTCCACAAATCAC
	R'	CTTCTCTTTGCAGATCCTGGTATA
ct223	F'	GTTGCTTTGGGAGCTGTTATTT
	R'	TGAGTTGCTTGTGAGCTTCTAT
clpP2	F'	GTTAGCGATTTACGACACCATTC
	R'	CCCTTTGTCCCTGCAGATAATA
pgp6	F'	CAAGAAGACATCCTAGCGGTAAA
	R'	AGTTGTTGAGGAGAAGTGATCC
hctB	F'	AACTGTAGCAGCTCGTAAGC
	R'	TTTGCGAGCTACAGTCTTCTT
