# Supplementary material for: Inclusion Membrane Growth and Composition Are Altered by Overexpression of Specific Inclusion Membrane Proteins in Chlamydia trachomatis L2
Source: Infect Immun. 2021 Jun 16;89(7):e00094-21. doi: 10.1128/IAI.00094-21 (PMC8208519; doi:10.1128/IAI.00094-21)
Supplement: Supplemental file 2 — Fig. S1 to S11. Download IAI.00094-21-s0002.pdf, PDF file, 5.88 MB [file iai.00094-21-s0002.pdf]

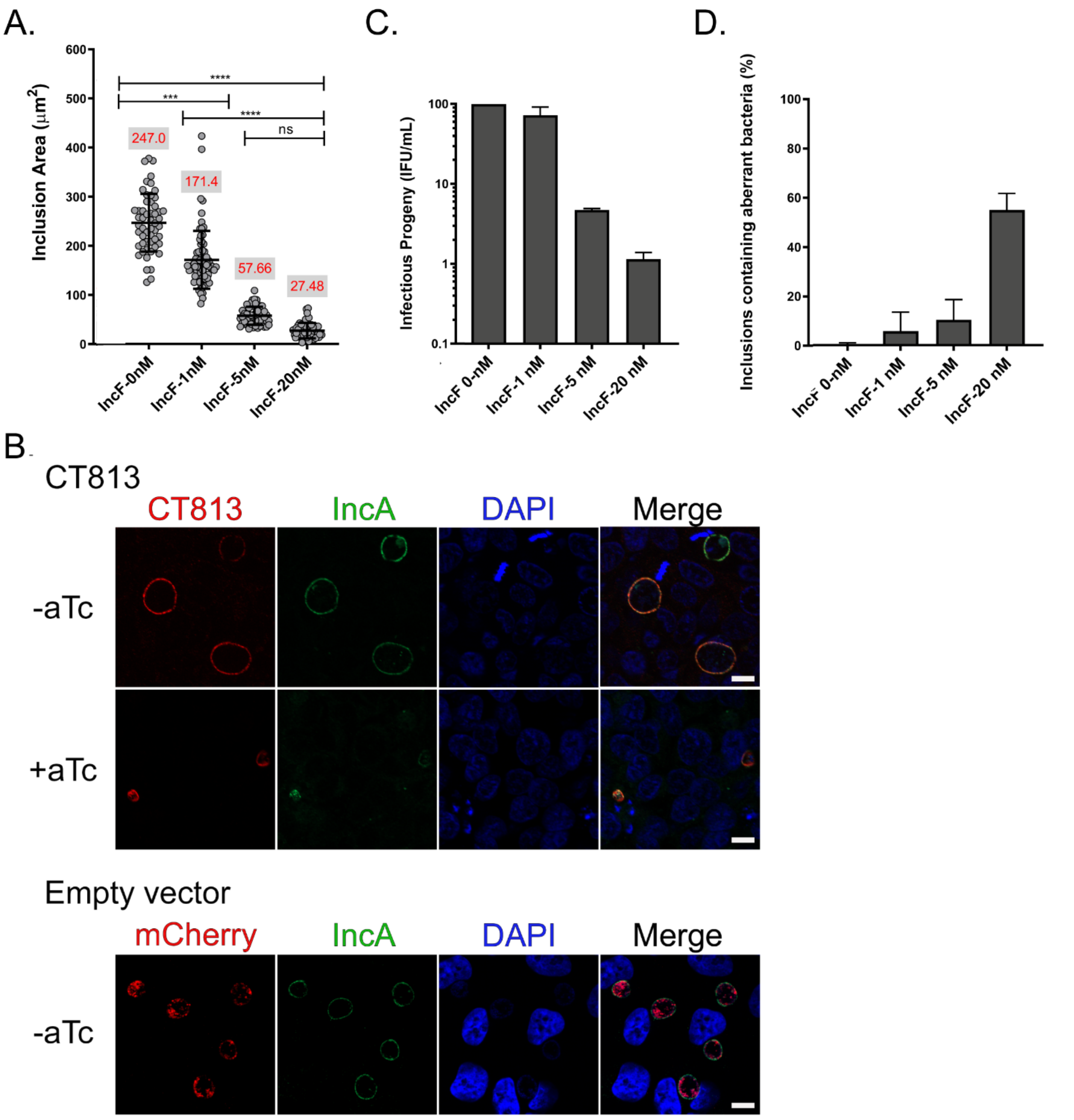

Fig. S1. The overexpression of IncF-FLAG and CT813 from *C. trachomatis* L2 transformed strains negatively impacts inclusion growth compared to L2 transformed with empty vector.

A) HeLa cells seeded on coverslips were infected with *C. trachomatis* L2 IncF-FLAG and induced at 7 hpi with 1, 5 or 20 nM aTc or not induced. Coverslips were methanol fixed at 36 hpi and stained for immunofluorescence to determine the inclusion area. A minimum of 100 inclusions per condition were measured using ImageJ. The inclusion area (μm^2) and the standard deviation was plotted using GraphPad Prism 8.4.0, and then analyzed for statistical significance by an ordinary

one-way ANOVA and Tukey's multiple comparisons test. These data are combined from 3 biological replicates.

B) HeLa cells infected with *C. trachomatis* L2 CT813 were induced at 7 hpi with 20 nM aTc or not induced. Coverslips were methanol fixed at 36 hpi and stained for immunofluorescence to observe construct expression (CT813; red), IncA (green), or DNA (DAPI; blue). For the empty vector control, HeLa cells were infected with L2 transformed with an pBOMB-mCherry that constitutively expresses mCherry. Coverslips were fixed at 24 hpi with glutaraldehyde/formaldehyde to preserve mCherry and stained for immunofluorescence to observe IncA (green), or DNA (DAPI; blue). For both transformed strains, coverslips were imaged at 63x magnification (Scale bar= 10 μm).

C) HeLa cells infected in duplicate with *C. trachomatis* L2 IncF-FLAG were induced for expression at 7 hpi using 1nM, 5 nM, or 20 nM aTc. At 36 hpi, infected monolayers were lysed, serially diluted, and infected onto a fresh monolayer of HeLa cells (i.e., secondary infection) in media containing penicillin to enumerate infectious progeny (Inclusion Forming Units (IFU)/mL). Infectious progeny (IFU/mL) (normalized to uninduced and expressed as a percentage of uninduced) from three biological replicates and the standard deviation were plotted and statistically analyzed as in A using GraphPad Prism 8.4.0. ****, $p<0.0001$. Only inclusions with wild-type phenotype were enumerated. Raw progeny data for *C. trachomatis* L2 IncF-FLAG 0 nM $5.70\times10^7 \pm 5.12\times10^7$ IFU/mL, IncF-FLAG 1 nM $4.93\times10^7 \pm 3.81\times10^7$ IFU/mL, IncF-FLAG 5 nM $2.77\times10^6 \pm 3.55\times10^6$ IFU/mL, and IncF-FLAG 20 nM $1.09\times10^5 \pm 6.94\times10^5$ IFU/mL.

D) Plasmid loss was indicated by inclusions containing aberrant bacteria in media containing penicillin (i.e., sensitivity due to the loss of the plasmid-encoded *bla*^{r}). To enumerate the percent of inclusions containing aberrant bacteria, the number of inclusions with aberrant bacteria was divided by the total number of inclusions counted (part C) from three biological replicates, and the average and standard deviation was plotted and statistically analyzed as in A and C using GraphPad Prism 8.4.0. ***, $p=0.0002$.

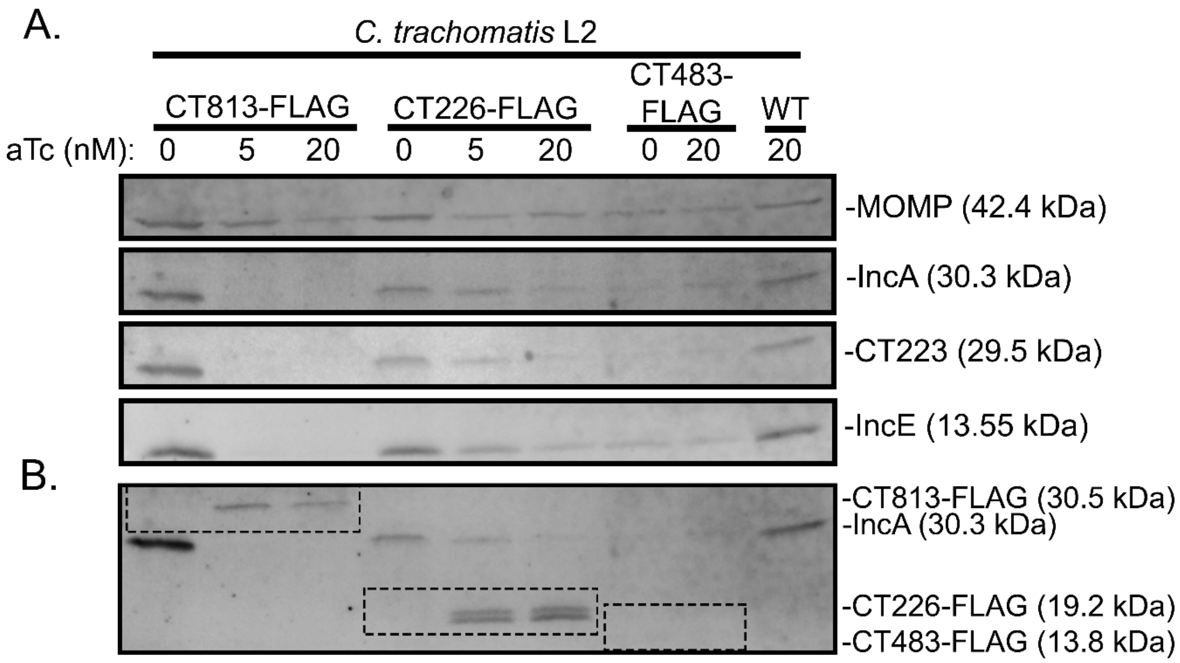

Fig. S2. The overexpression of CT813-FLAG from *C. trachomatis* L2 results in decreased detectable IncA, CT223, and IncE at 36 hpi.

HeLa cells were infected with *C. trachomatis* L2 CT813-FLAG, CT226-FLAG, CT483-FLAG transformed strains, or wild-type *C. trachomatis* L2, and either not induced or induced at 7 hpi (5 nM or 20 nM aTc). Infected monolayers were lysed in RIPA buffer modified with 1% Triton X-100, HALT protease inhibitor with EDTA, Clasto-lactacystin β-lactone, and nuclease. Normalized lysates were separated by SDS-PAGE and transferred to PVDF to blot for chlamydial proteins. A) western blot probing for MOMP, IncA, CT223, and IncE (as indicated). B) western blot for Inc-FLAG constructs (and IncA). Dotted lines indicate the lanes for each transformed strain at the expected molecular weight. These data are representative of 3 independent experiments.

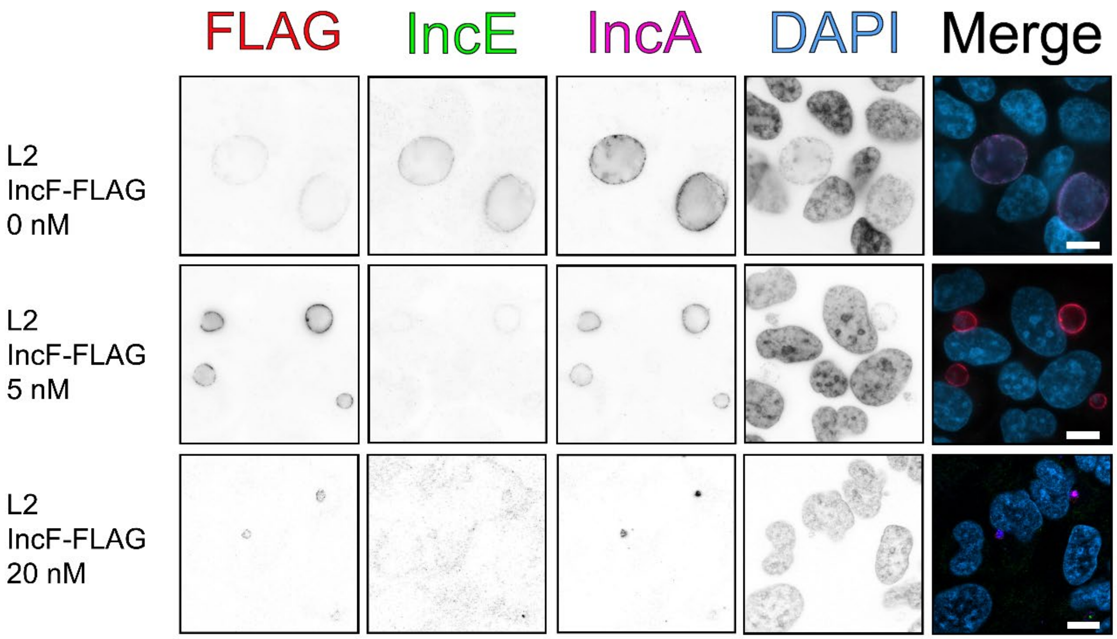

Fig. S3. The overexpression of IncF-FLAG from *C. trachomatis* L2 IncF-FLAG results in the loss of endogenous IncE in the inclusion membrane.

A) HeLa cells infected with *C. trachomatis* L2 IncF-FLAG were induced with 5 or 20 nM aTc, or not induced at 7 hpi. Coverslips were methanol fixed at 36 hpi and stained for immunofluorescence to observe expression of the constructs (FLAG; red), IncE (green), IncA (pink), or DNA (DAPI; blue). Coverslips were imaged at 63x magnification using the same exposure time when measuring Inc intensity for each sample. Scale bar= 10 µm. Individual panels were converted to black and white and inverted for visualization of the loss of IncE staining.

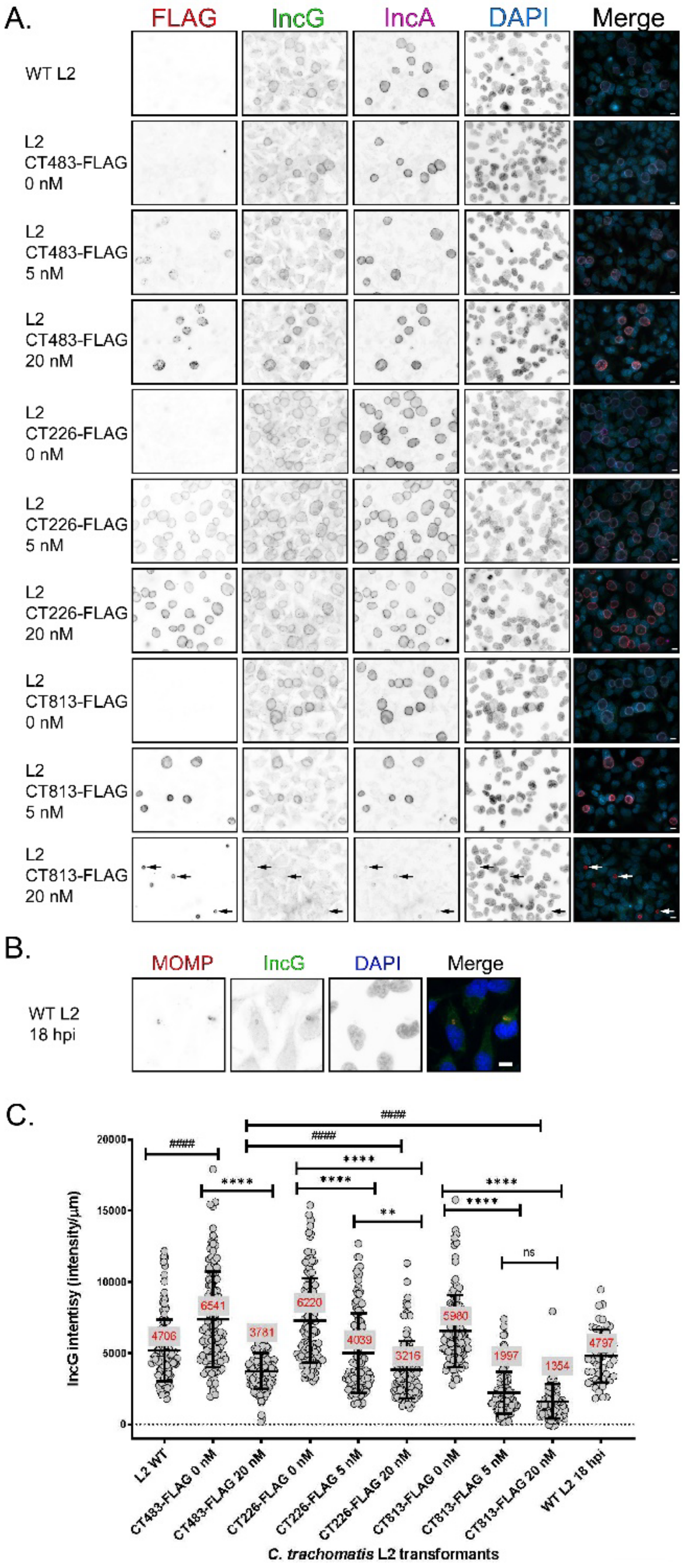

A.
FLAG
IncG
IncA
DAPI
Merge
WT L2
L2 CT483-FLAG 0 nM
L2 CT483-FLAG 5 nM
L2 CT483-FLAG 20 nM
L2 CT226-FLAG 0 nM
L2 CT226-FLAG 5 nM
L2 CT226-FLAG 20 nM
L2 CT813-FLAG 0 nM
L2 CT813-FLAG 5 nM
L2 CT813-FLAG 20 nM
B.
MOMP
IncG
DAPI
Merge
WT L2 18 hpi
C.
IncG intentisy (intensity/µm)
20000
15000
10000
5000
0
####
####
####
****
****
****
**
****
****
ns
4706
6541
3781
6220
4039
3216
5980
1997
1354
4797
L2 WT
CT483-FLAG 0 nM
CT483-FLAG 20 nM
CT226-FLAG 0 nM
CT226-FLAG 5 nM
CT226-FLAG 20 nM
CT813-FLAG 0 nM
CT813-FLAG 5 nM
CT813-FLAG 20 nM
WT L2 18 hpi
C. trachomatis L2 transformants

D.

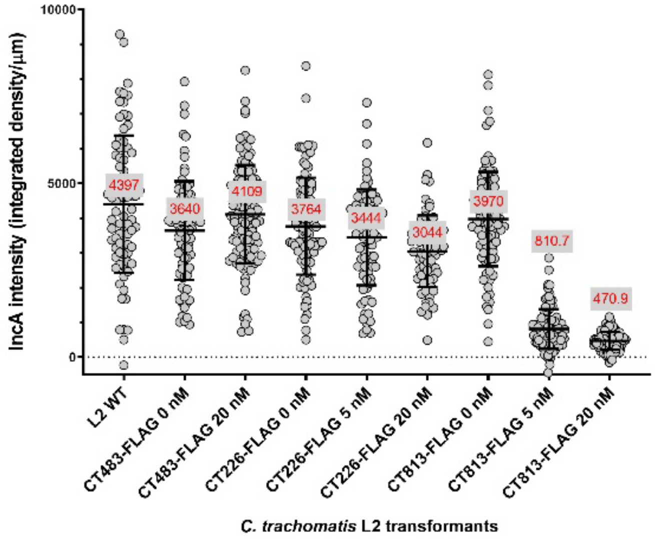

Fig. S4. The overexpression of CT813-FLAG, but not CT226-FLAG, or CT483-FLAG from *C. trachomatis* L2 transformed strains results in loss of endogenous IncG and reduced IncA intensity.

A) HeLa cells infected with *C. trachomatis* L2 IncF-FLAG were induced with 5 or 20 nM aTc (or not induced) at 7 hpi. Coverslips were methanol fixed at 36 hpi and stained for immunofluorescence to observe expression of the constructs (FLAG; red), IncG (green), IncA (pink), or DNA (DAPI; blue). Coverslips were imaged using the same exposure for each sample at 63x magnification. Scale bar= 10 µm. Individual panels were converted to black and white and inverted to visualize the loss of IncG staining. Arrows indicate inclusions lacking IncG. These data are representative of 3 independent experiments.

B) *C. trachomatis* L2 infected HeLa cells were fixed at 18 hpi and stained for immunofluorescence to observe IncE (green) and DNA (blue). Coverslips were imaged as in A using the same exposure time for each sample at 63x magnification. Scale bar= 10 µm.

C) The intensity of IncG, or IncA (D) at the inclusion membrane was measured with ImageJ from images in part A. The background integrated density was subtracted from individual images and the intensity was normalized to the perimeter of individuals inclusions (integrated density/µm) and standard deviation were plotted using GraphPad Prism 8.4.0. Samples were analyzed for statistical significance using an ordinary one-way ANOVA and Tukey's multiple comparisons test. A minimum of 80 inclusions were measured per condition for three independent experiments. The mean integrated density/µm is reported in red for each sample measured. Asterisks denote statistical significance, where **** p< 0.0001 between *C. trachomatis* L2 transformed strains and #### indicates p< 0.0001 between *C. trachomatis* L2 transformed strains.

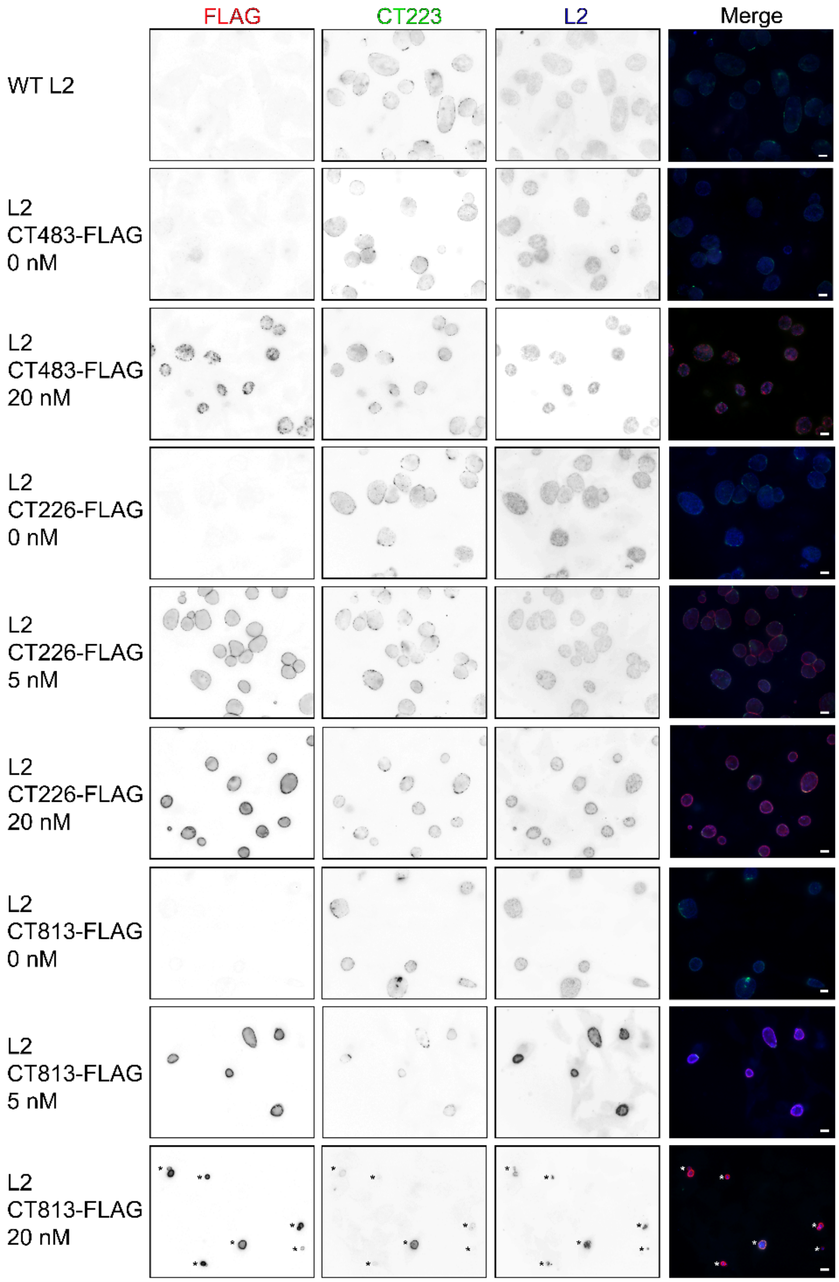

Fig. S5. The overexpression of CT813-FLAG, but not CT226-FLAG, or CT483-FLAG from *C. trachomatis* L2 transformed strains results in loss of endogenous CT223.

HeLa cells infected with *C. trachomatis* L2 IncF-FLAG were induced with 5 or 20 nM aTc (or not induced) at 7 hpi. Coverslips were methanol fixed at 36 hpi and stained for immunofluorescence to observe expression of the constructs (FLAG; red), CT223 (green), and chlamydiae (L2; blue). Coverslips were imaged using the same exposure time for each sample at 63x magnification. Scale bar= 10 µm. Individual panels were converted to black and white and inverted to visualize the loss of CT223. Asterisks indicate CT813-FLAG positive inclusions. These data are representative of 2 independent experiments.

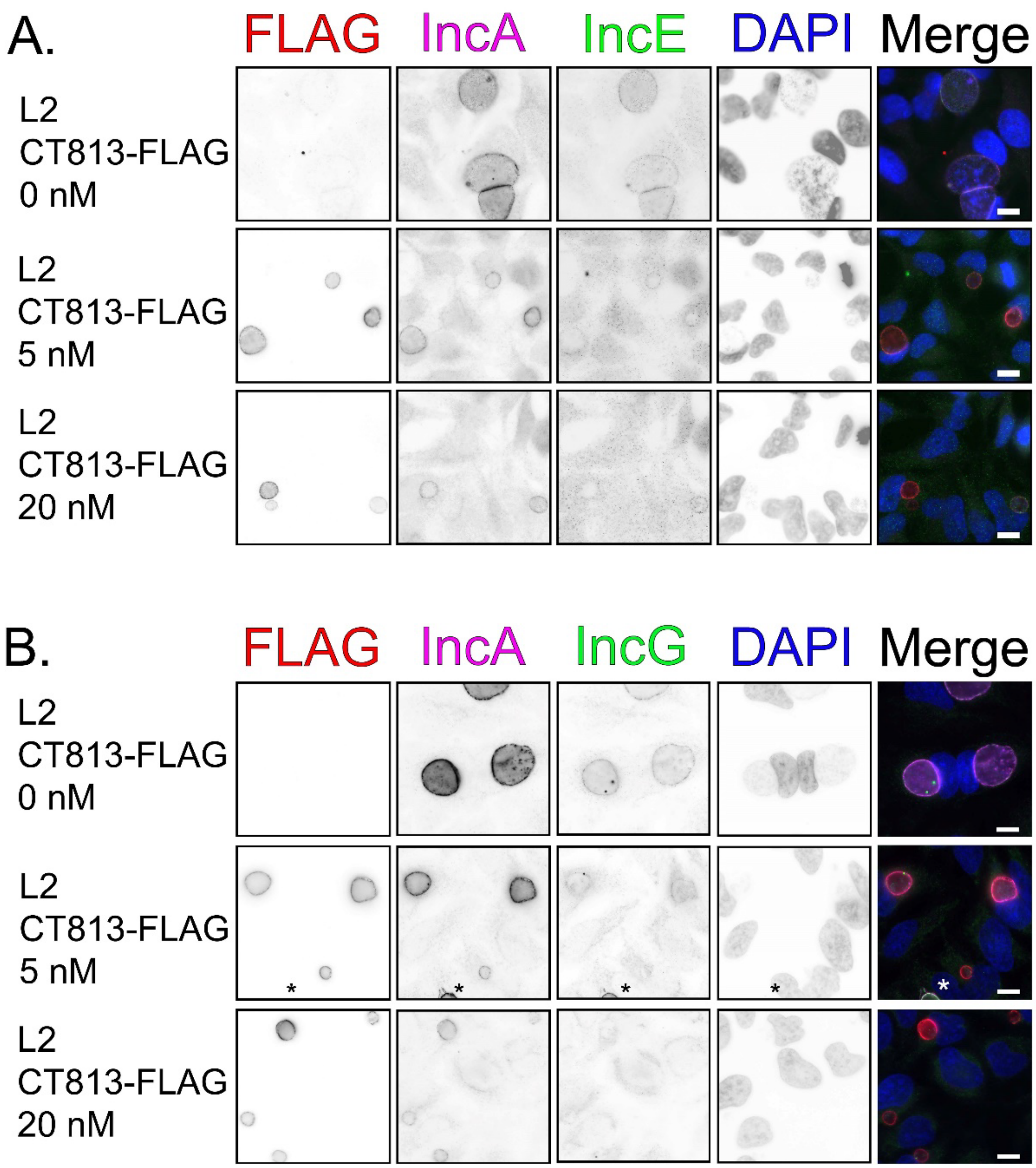

Fig. S6. Induction of CT813-FLAG from *C. trachomatis* L2 CT813-FLAG at 14.5 hpi results in the loss of both IncE and IncG in the inclusion membrane.

Hela cells infected with *C. trachomatis* L2 CT813-FLAG were either not induced or induced using 5 or 20 nM aTc at 14.5 hpi. Coverslips were methanol fixed at 36 hpi and stained for indirect immunofluorescence to detect the construct expression (FLAG; red), IncA (pink) DNA (DAPI; blue), and either IncE (top panel A; green) or IncG (bottom panel B; green). Coverslips were imaged using Zeiss without Apotome at 63x magnification. Asterisks (*) indicate inclusions with aberrant bacteria that have IncA and IncG at the inclusion membrane (scale bar=10 µm). These data are representative of 3 independent experiments.

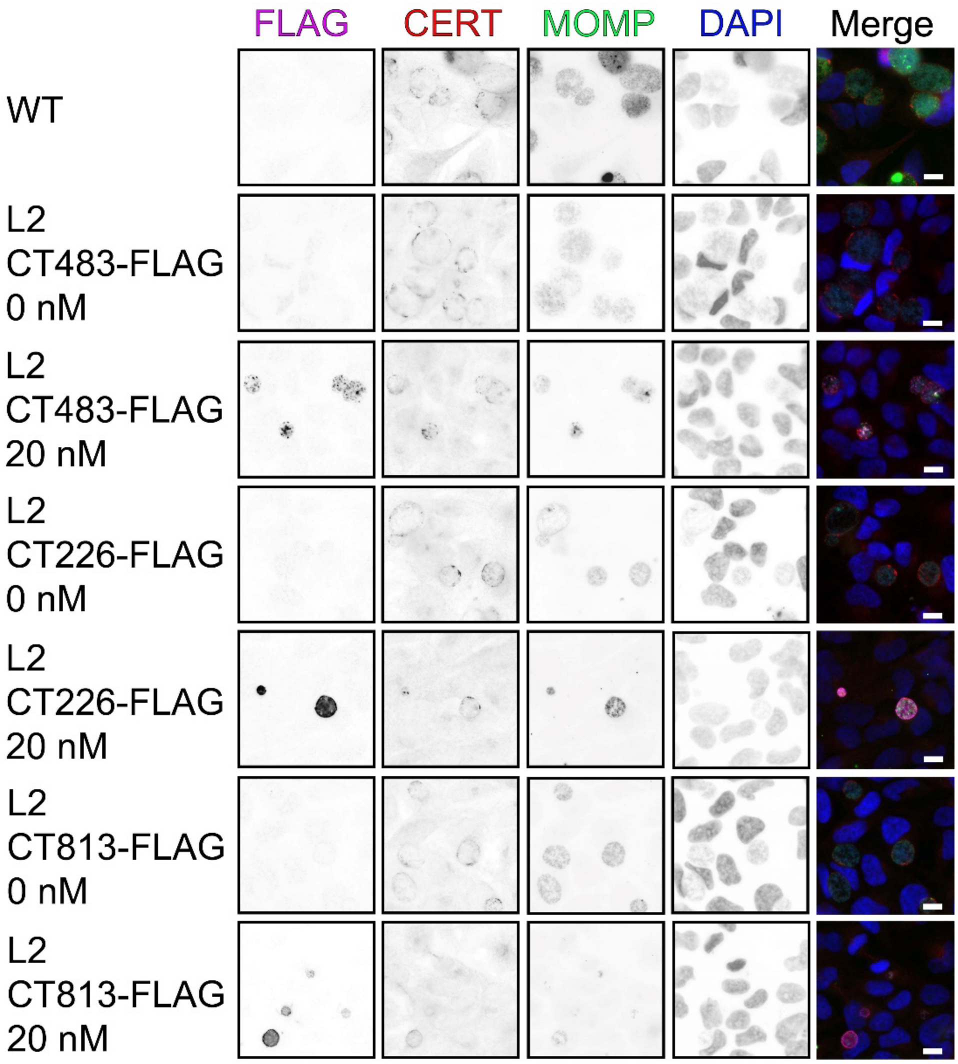

Fig S7. The localization of CERT at the inclusion membrane is not impacted by the overexpression of CT813-FLAG from *C. trachomatis* L2.

HeLa cells infected with *C. trachomatis* L2 transformed strains or wild-type L2 were induced at 7 hpi with 5 or 20 nM aTc, or not induced. Coverslips were fixed in 2% paraformaldehyde at 36 hpi, permeabilized with 0.5% triton X-100, and stained for immunofluorescence to observe expression of the Inc-FLAG constructs (FLAG; pink), CERT (red), MOMP (green), or DNA (DAPI; blue). Coverslips were imaged at 63x magnification using the same exposure (scale bar= 10 µm). These data are representative of 3 independent experiments.

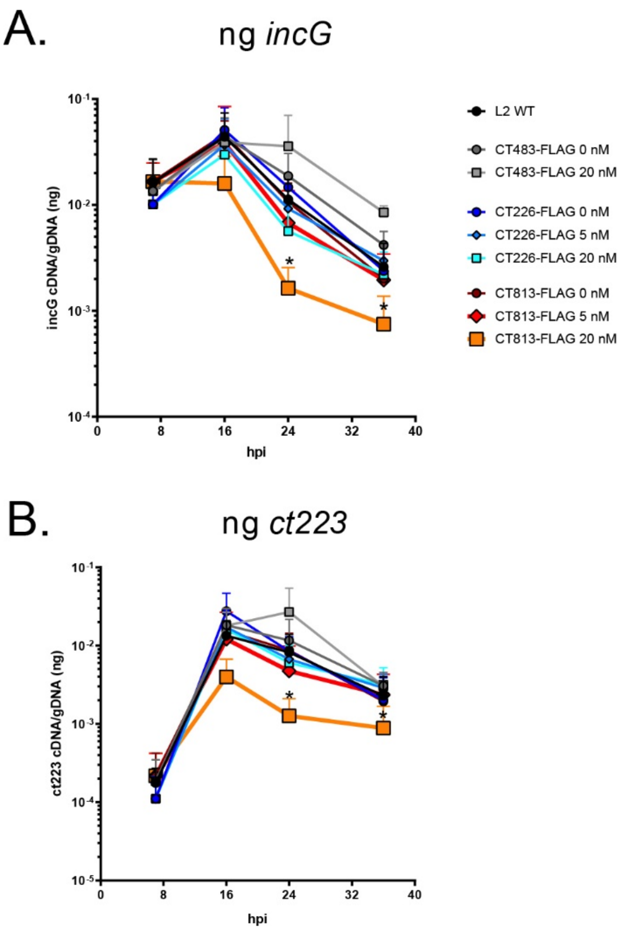

Fig S8. The overexpression of CT813-FLAG from *C. trachomatis* L2 CT813-FLAG results in reduced transcription of the early gene, *incG,* and mid-cycle gene, *ct223.*

HeLa cells were infected with *C. trachomatis* L2 CT813-FLAG, CT226-FLAG, CT483-FLAG transformed strains, or wild-type *C. trachomatis* L2, and either not induced or induced at 7 hpi (5 nM or 20 nM aTc). RNA and DNA were collected from separate wells of a 6-well plate at 7, 16, 24, and 36 hpi. Normalized RNA was reverse transcribed to cDNA, and *inc* expression was measured by quantitative PCR. cDNA (ng) was normalized to genomic DNA (ng) for three biological replicates and then these data were plotted using GraphPad Prism 8.4.0. Transcript profile of A) the early *inc, incG* and B) mid-cycle *inc,* ct223. Normalized transcripts from wild-type and *C. trachomatis* L2 CT813-FLAG were log transformed and then analyzed for statistical significance using a paired two-tailed students *t*-test. Asterisks denote statistical significance, where * p≤0.05.

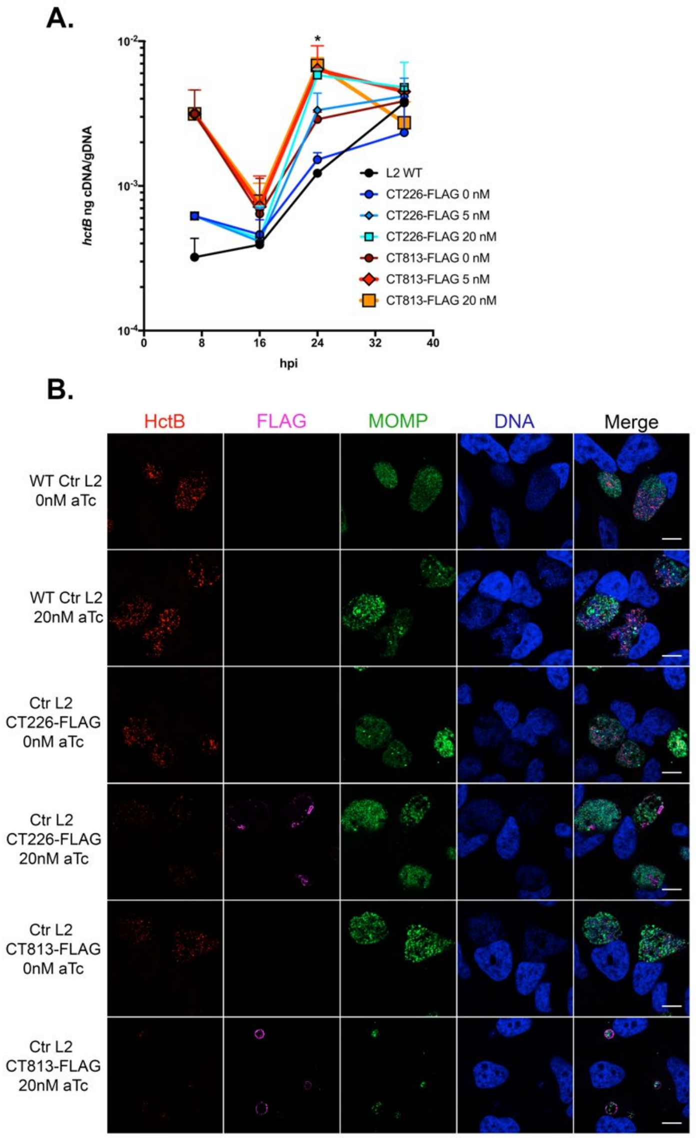

Fig. S9. *hctB* transcripts increase 24 hours post-infection after overexpression of CT813-FLAG,

HeLa cells were infected with *C. trachomatis* L2 CT813-FLAG, CT226-FLAG, CT483-FLAG transformed strains, or wild-type *C. trachomatis* L2, and either not induced or induced at 7 hpi (5 nM or 20 nM aTc). A) RNA and DNA were collected from separate wells of a 6-well plate at 7, 16, 24, and 36 hpi. RNA was reverse transcribed to cDNA, and *hctB* expression was measured by quantitative PCR. cDNA (ng) was normalized to genomic DNA (ng) for two biological replicates and then these data were plotted using GraphPad Prism 8.4.0. Transcript profile of *hctB*. Normalized transcripts from wild-type and *C. trachomatis* L2 CT813-FLAG were log transformed and then analyzed for statistical significance using a paired two-tailed students *t*-test. Asterisks denote statistical significance, where * $p \leq 0.05$. B) cells were fixed at 36 hpi and processed for indirect immunofluorescence to detect HctB (red), construct expression (FLAG; purple), chlamydial organisms (MOMP; green), and DNA (DAPI; blue). Samples were imaged on the Zeiss Axiovert at 63x magnification (scale bar = 10 μm).

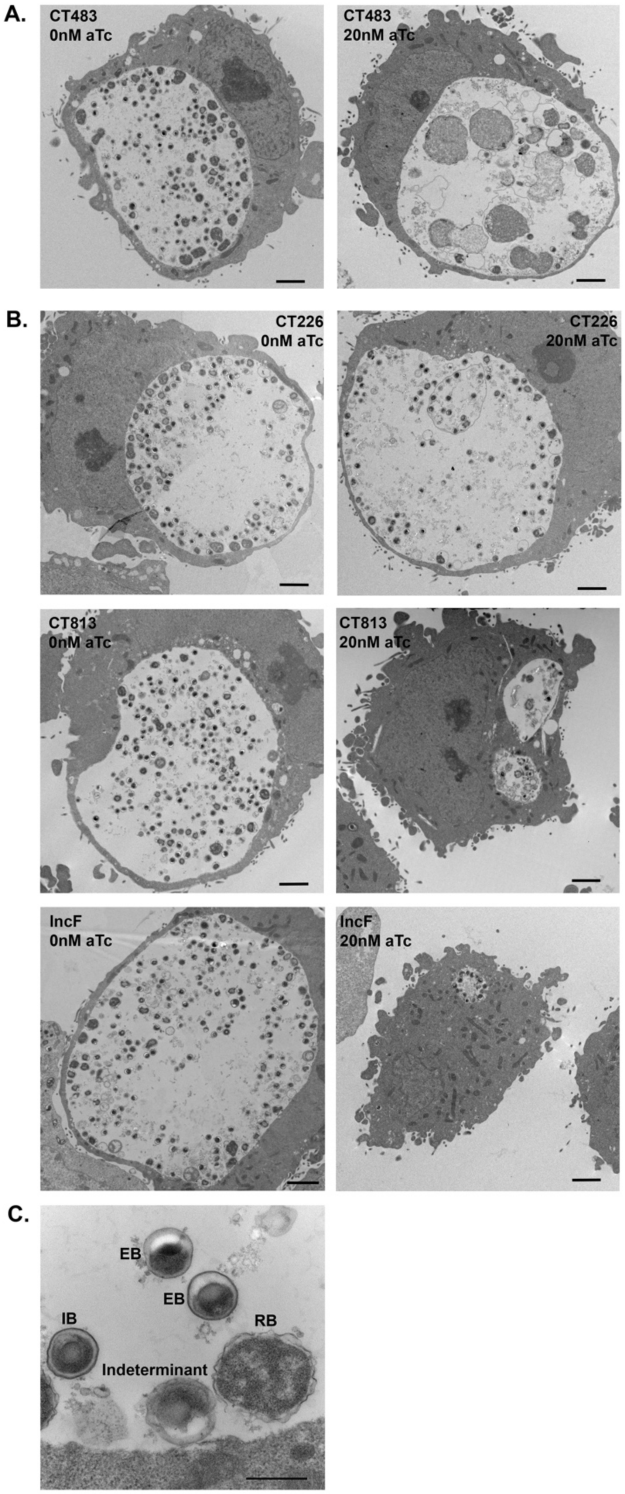

Fig. S10. Ultrastructural analysis of inclusions after the overexpression of CT483-FLAG, CT226-FLAG, CT813-FLAG, and IncF-FLAG from transformed chlamydial strains.

HeLa cells infected with *C. trachomatis* L2 transformed strains were either not induced or induced using 20 nM aTc at 7 hpi. At 36 hpi, HeLa cells were trypsinized, pelleted, and washed

with PBS, then resuspended in 2% glutaraldehyde and 2% formaldehyde in 0.1 M Phosphate buffer and processed by the UNMC electron microscopy core. Representative images from two technical replicates are shown. In A) overexpression of CT483-FLAG causes abnormally large forms, which is consistent with CT483 being a chlamydial membrane protein. In B) remaining transformed chlamydial strains that were uninduced or induced with 20nM aTc are shown. In C, examples of chlamydial developmental forms that were enumerated are shown. All samples were imaged using examined using a Tecnai G2 Spirit (FEI) TEM at (A and B) 4400x magnification with scale bar equal to 2 μm or (C) 52000x magnification with scale bar equal to 500 nm.

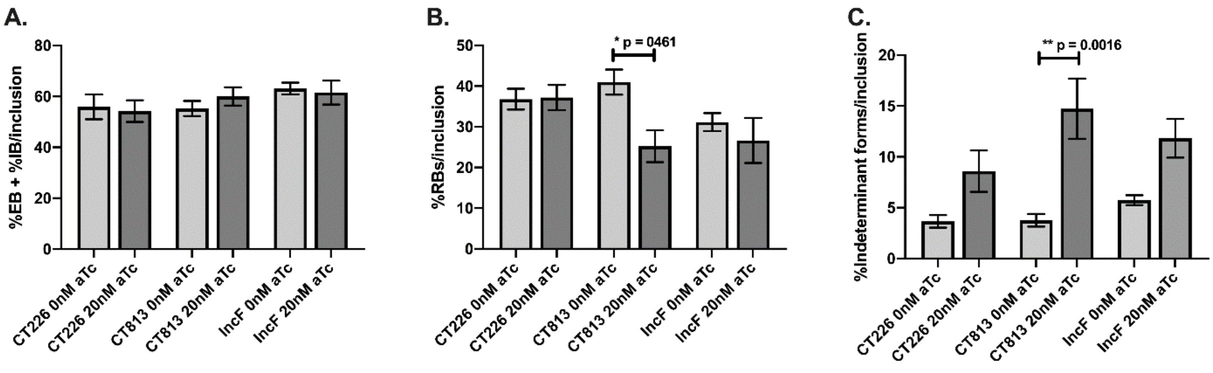

Fig. S11. Ultrastructural analysis of chlamydial morphological forms after overexpression of CT226-FLAG, CT813-FLAG, and IncF-FLAG from transformed chlamydial strains.

The chlamydial morphological forms in TEM images as seen in representative images in Fig. S7 B and C were enumerated. Data are represented as %EBs +%IBs/inclusion (A), %RBs/inclusion (B), and %Indeterminant forms/inclusion (C). The graphed results are means with standard error of the mean and are combined data from 2 technical replicates with the total number of inclusions analyzed: 14 (CT226 0nM aTc), 12 (CT226 20nM aTc), 18 (CT813 0nM aTc), 27 (CT813 20nM aTc), 20 (IncF 0nM aTc), and 23 (IncF 20nM aTc). The data were statistically analyzed in GraphPad Prism (v 8.4.3) using an ordinary One-Way Anova with Tukey’s multiple comparison test.
